# *Porphyromonas gingivalis* Suppresses Interferon‐Gamma Signaling in Macrophages Through a Contact‐Dependent, Gingipain‐Mediated Mechanism

**DOI:** 10.1002/mbo3.70290

**Published:** 2026-04-12

**Authors:** Shotaro Abe, Jun Ohshima, Masayoshi Morita, Nobutake Tanaka, Mikako Hayashi

**Affiliations:** ^1^ Department of Restorative Dentistry and Endodontology, Graduate School of Dentistry The University of Osaka Suita Osaka Japan

**Keywords:** bacterial secretion systems, immune evasion, interferon, macrophage activation, *Porphyromonas gingivalis*, STAT1 transcription factor

## Abstract

Interferon signaling serves as a crucial defense mechanism for host cells against intracellular pathogens. Interferon‐gamma (IFN‐γ) binding to macrophages impacts the expression of approximately 2000 genes, activating them to enhance intracellular bactericidal activity. While various immune evasion strategies of *Porphyromonas gingivalis* have been extensively studied, the specific mechanisms by which it suppresses IFN‐γ‐mediated macrophage activation remain insufficiently characterized. In this study, we elucidated the molecular mechanism by which *P. gingivalis* suppresses interferon signaling in macrophages, with a particular focus on *STAT1* transcript abundance, because *STAT1* encodes a central transcription factor in the IFN‐γ pathway. RNA‐seq analysis revealed that *P. gingivalis* infection reduced the mRNA abundance of approximately 41% of genes upregulated following IFN‐γ stimulation, including *STAT1* transcripts and other interferon‐related genes. Further experiments showed that direct contact between the bacterium and host cells is necessary for this inhibition. This process involves the Type IX Secretion System (T9SS) and gingipains. Notably, strains lacking all gingipains (Kgp, RgpA, and RgpB) failed to suppress *STAT1* transcript abundance and instead allowed nuclear translocation of phosphorylated STAT1. These gingipain‐deficient strains also exhibited reduced invasive ability, correlating with their diminished capacity to suppress interferon signaling and macrophage activation. In conclusion, our findings demonstrate that *P. gingivalis* inhibits interferon signaling in macrophages through intracellular infiltration, with T9SS and gingipains playing essential roles in this immunosuppressive mechanism. These results provide valuable insights into the immune evasion strategies of *P. gingivalis* and suggest potential therapeutic targets for combating periodontopathic diseases.

## Graphical abstract

This study reveals that *Porphyromonas gingivalis* suppresses IFN‐γ signaling by downregulating STAT1 in macrophages. Gingipain protease activity and the Type IX Secretion System are essential for this immune evasion mechanism. These findings identify a novel bacterial strategy for host immune suppression and suggest new therapeutic targets for *P. gingivalis*‐associated diseases.

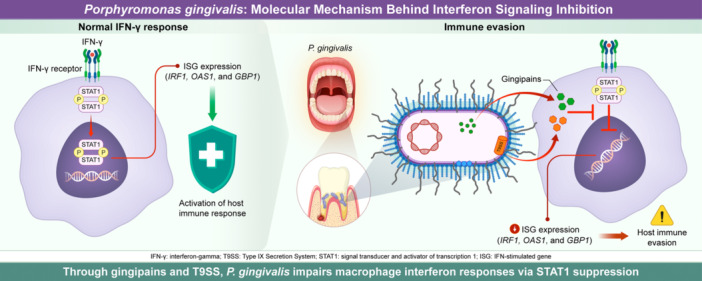

## 1 Introduction

Humans and bacteria coexist in a delicate balance that, when disrupted, can lead to significant health issues (Suzuki and Ley 2020). Changes in the indigenous bacterial flora have been reported to influence various host functions, including immune responses, metabolism, and inflammatory processes. Periodontal disease, an oral infection affecting 10%–15% of the global population, is one example where shifts in the composition of the oral microbiota play a critical role in disease progression (Paster et al. 2001). When a host's immune system is weakened, bacterial virulence factors become dominant, disrupting this balance and increasing the risk of systemic health complications (Hajishengallis 2015).

The oral cavity is home to a diverse microbial community, housing between 500 and 700 bacterial species and totaling over 6 billion bacteria (Deo and Deshmukh 2019). Maintaining balance within this microbiota is vital for oral and systemic health. Among these bacteria, *Porphyromonas gingivalis* (*P. gingivalis*) has been identified as a key pathogen capable of disrupting the microbial balance or inducing dysbiosis, by suppressing the host's immune system (Maekawa et al. 2014). This pathogen not only causes inflammation of the gums and surrounding tissues, leading to tooth loss, but also transitions the microbiota to a highly pathogenic state, even when present in small quantities (Hajishengallis et al. 2011; Abusleme et al. 2013; Hajishengallis 2014). *P. gingivalis* is recognized as a major contributor to periodontal disease and has been increasingly implicated in systemic diseases (Hajishengallis and Chavakis 2021), such as cardiovascular disease (Pussinen et al. 2003), Alzheimer's disease (Dominy et al. 2019), and pancreatic and colorectal cancers (X. Wang et al. 2021; Tan et al. 2022). Its ability to invade host cells allows it to evade immune detection, leading to persistent infections that elevate the risk of these diseases (M. Wang et al. 2010; Dickinson et al. 2011).

At the forefront of the immune response to infections, innate immune cells play a pivotal role in orchestrating the initial defense mechanisms. Among these, macrophages are crucial components of the inflammatory response. They localize to infection sites, engulf pathogens through phagocytosis, and present pathogen‐derived antigens to activate other immune cells (Thakur et al. 2019; Kolliniati et al. 2022). Furthermore, the activation of macrophages is strongly enhanced by interferon‐gamma (IFN‐γ) produced upon antigen presentation, leading to the promotion of innate and adaptive immune responses (Schoenborn and Wilson 2007; Hu and Ivashkiv 2009). Thus, the inhibition of IFN signaling provides an effective means for *P. gingivalis* to evade detection by the host immune system and survive intracellularly (Jauregui et al. 2013; Rodriguez‐Hernandez et al. 2021). Despite growing evidence of the importance of IFN signaling suppression in host–pathogen interactions, the precise molecular mechanisms by which *P. gingivalis* achieves this remain poorly understood. It is unclear how specific bacterial factors contribute to IFN signaling inhibition and how this suppression facilitates intracellular survival and immune evasion.

This study aims to elucidate the molecular mechanisms by which *P. gingivalis* suppresses IFN signaling, identifying the key factors involved, and exploring how they contribute to immune evasion and intracellular survival. By addressing these questions, this research seeks to uncover new aspects of *P. gingivalis* pathogenicity and identify potential therapeutic targets for infection control and treatment of associated diseases. Furthermore, focusing on specific mechanisms of IFN signaling inhibition, which have not been addressed in previous studies, will provide deeper insights into the dynamic interactions between host and pathogen.

## 2 Materials and Methods

### 2.1 Host Cell Culture

In this study, we used several cell lines, including the human monocytic leukemia cell line THP‐1 (ATCC, Manassas, VA, USA), human leukemia cell line K562 (JCRB Cell Bank, Osaka, Japan), human oral epithelial cell line Ca9‐22 (JCRB Cell Bank), human fibrosarcoma cell line HT‐1080 (JCRB Cell Bank), human osteosarcoma U2OS (JCRB Cell Bank), human cervical cancer cell line HeLa (JCRB Cell Bank), and human gingival cell line TIGK (ATCC).

THP‐1 and K562 cell cultures were maintained in RPMI 1640 medium (Nacalai Tesque, Kyoto, Japan) supplemented with 100 U/mL penicillin, 0.1 mg/mL streptomycin (Nacalai Tesque), and 10% fetal bovine serum (FBS; Nichirei Bioscience, Tokyo, Japan) at 37°C with 5% CO_2_ and 100% humidity. To differentiate THP‐1 cells into macrophage‐like cells, phorbol‐12‐myristate‐13‐acetate (PMA; Sigma Aldrich, St. Louis, MO, USA) was added at a final concentration of 100 nM. The culture medium was changed after 48 h.

Ca9‐22, HT‐1080, U2OS, and HeLa cells were cultured in Dulbecco's modified Eagle Medium (Nacalai Tesque) supplemented with 100 U/mL penicillin, 0.1 mg/mL streptomycin (Nacalai Tesque) and 10% FBS (Nichirei Bioscience). TIGK cells were cultured using Dermalife Basal Medium (Lifeline, Walkersville, MD, USA) supplemented with DermaLife K LifeFactors Kit (Lifeline), a growth factor cocktail for epithelial cell culture. All cell cultures were maintained at 37°C with 5% CO_2_ and 100% humidity throughout the experimental period.

### 2.2 Bacterial Strains Used and Culture Conditions

In this study, we used the following strains of *P. gingivalis*: ATCC 33277, ATCC BAA‐308 (W83), and ATCC BAA‐3229 (W50) (Table S1). The cultures were grown under anaerobic conditions in GAM medium (Nissui, Hiroshima, Japan) supplemented with 5 μg/mL hemin (Sigma Aldrich) and 1 μg/mL menadione (Wako, Osaka, Japan). Antibiotic‐resistant mutants were cultured in the medium containing erythromycin (Wako) at a final concentration of 10 μg/mL, tetracycline (Wako) at 0.7 μg/mL, or chloramphenicol (Wako) at 3 μg/mL, as required.

### 2.3 Bacterial Infection of Cell Lines

The human monocytic leukemia cell line THP‐1 was treated with PMA for 48 h, followed by the addition of IFN‐γ (PeproTech, Rocky Hill, NJ, USA) at a final concentration of 20 ng/mL to the differentiated macrophage‐like cells for 24 h to stimulate their activation. Subsequently, *P. gingivalis* was used to infect cells, or LPS‐PG Ultrapure (InvivoGen, San Diego, CA, USA), a purified lipopolysaccharide (LPS) from *P. gingivalis* and a Toll‐like receptor ligand, was added at a concentration of 5 μg/mL. For other cells (K562, TIGK, Ca9‐22, HT‐1080, U2OS, HeLa), IFN‐γ was applied at the time of seeding into multi‐well plates, and *P. gingivalis* infection was conducted 24 h later. *P. gingivalis* was harvested from 5 mL of liquid culture at the log phase by centrifugation (2330*g*, 10 min), washed three times with PBS (Nacalai Tesque), and resuspended in 1 mL of PBS. The turbidity of the bacterial suspension was measured using a colorimeter (Colourwave CO7500; Biochrom, Waterbeach, Cambridge, UK), and the suspension was adjusted to prepare inocula corresponding to the desired multiplicity of infection (MOI) when applied to each cell line. Noninfected samples received an equal volume of PBS. For each condition, three independent biological replicates were prepared.

### 2.4 Total RNA Extraction

Cellular total RNA was extracted using the FastGene RNA Basic Kit (NIPPON Genetics, Tokyo, Japan). Bacterial total RNA was extracted using the RNAprotect Bacteria Reagent (QIAGEN, Hilden, Germany) and the RNeasy Mini Kit (QIAGEN) according to the manufacturer's instructions.

### 2.5 RNA Sequencing

Total RNA extracted from three groups of samples, namely human macrophage‐like cells THP‐1 activated by IFN‐γ for 24 h (IFN‐γ group), samples subsequently infected with *P. gingivalis* ATCC 33277 strain (wild‐type (WT)) (IFN‐γ + *P. gingivalis* group), and untreated samples (control group), was subjected to RNA‐seq analysis. IFN‐γ was applied at a final concentration of 20 ng/mL. Infection with *P. gingivalis* was performed as described in Section 2.3, and infected cells were harvested 24 h postinfection. For each condition, three independent biological replicates were prepared. The extracted total RNA samples were sent to Azenta (Tokyo, Japan) for Bioanalyzer quality control analysis (Agilent, Santa Clara, CA, USA) and next‐generation sequencing. Strand‐specific cDNA libraries were prepared from each sample's total RNA following the manufacturer's protocol. The libraries were then sequenced on the Illumina HiSeq platform, generating 20–23 million paired‐end (PE) sequence reads of 150 bp per sample. Subsequently, the PE FASTQ files were trimmed using the FASTX‐Toolkit software. The trimmed sequence reads were mapped using HISAT2, and gene‐level read counts were determined using Cufflinks. FPKM, FPKM‐UQ, and TPM values were calculated based on these counts. These data were combined with the web tools iDEP (http://bioinformatics.sdstate.edu/idep96/) and Venn Diagram (https://bioinformatics.psb.ugent.be/webtools/Venn/), and additional bioinformatics analysis software in RaNA‐seq (https://ranaseq.eu/) was used for further analysis (Hulsen et al. 2008; Ge et al. 2018; Prieto and Barrios 2020). For visualization, normalized expression values were summarized across biological replicates within each condition as indicated in the corresponding figure legends.

### 2.6 Quantitative Real‐Time PCR (RT‐qPCR)

cDNA was synthesized from the obtained RNA by a High‐Capacity RNA‐to‐cDNA Kit (Thermo Fisher Scientific, Waltham, MA, USA) according to the manufacturer's instructions. Quantitative RT‐PCR was performed on a 7300 Fast Real‐Time PCR System (Thermo Fisher Scientific) with Power SYBR Green Master Mix (Thermo Fisher Scientific). Relative expression levels were calculated using the ΔΔCt method. For host‐cell gene expression analysis, the data were normalized to human *GAPDH*. For bacterial gene expression analysis, the data were normalized to *P. gingivalis* 16S rRNA. The primer sequences are listed in Table S2.

### 2.7 Transwell Assay

THP‐1 cells were seeded at a density of 1 × 10^6^ cells/mL in 6‐well multi‐well plates and treated with PMA for 48 h to induce differentiation into macrophage‐like cells. A transwell with a 0.4 μm pore size (FALCON, Franklin Lakes, NJ, USA) was placed on top of a 6‐well multi‐well plate, and *P. gingivalis* (MOI = 100) was added to the transwell, ensuring contact with the cell culture medium.

### 2.8 PFA and Heat Treatment of *P. gingivalis*

Prior to infection experiments, washed *P. gingivalis* cells were fixed in 4% paraformaldehyde (PFA) in 1 mL of PBS for 10 min. Following PFA fixation, cells were washed three times with PBS to remove residual fixative. For the heat treatment, the washed *P. gingivalis* organisms were incubated at 70°C for 30 min. For Tosyl‐l‐lysyl‐chloromethane hydrochloride (TLCK) treatment, the washed *P. gingivalis* cells were incubated with 1 mL of PBS containing 100 µM TLCK (Nacalai Tesque) for 30 min under anaerobic conditions. Then, the cells were washed with PBS and used for infection (Cremer et al. 2009).

### 2.9 Plasmid Construction and Generation of Mutant Strains

Gene mutants of *P. gingivalis* were generated as previously described (Jung et al. 2019). The upstream (UA_F/UA_R) and downstream (LA_F/LA_R) sequences of the target genes were amplified using the chromosomal DNA of *P. gingivalis* ATCC 33277 as a template (primer set used: Table S3). Additionally, the erythromycin resistance gene was amplified from pG106‐KA (Plasmid #178041: Addgene, Watertown, MA, USA). Each DNA fragment was treated with restriction enzymes and inserted into the cloning plasmid pCR‐Blunt vector (Zero Blunt PCR Cloning Kit: Thermo Fisher Scientific) using a Quick Ligation Kit (New England BioLabs, Ipswich, MA, USA). The plasmid was used as a template, and the upstream and downstream primers (UA_F, LA_R) were used for amplification. The resulting DNA fragments were introduced into *P. gingivalis* ATCC 33277 by electroporation to generate a gene‐deficient strain through homologous recombination.

### 2.10 Observation of Colony Pigment on Blood Agar

*P. gingivalis* was inoculated onto Anero Columbia RS blood agar (Nippon Becton Dickinson, Tokyo, Japan) and incubated under anaerobic conditions until colonies were visible. After incubation, the pigmentation of the formed colonies was observed visually.

### 2.11 Hemagglutination Assay

*P. gingivalis* was cultured under anaerobic conditions, washed three times with PBS, and resuspended in 1 mL of PBS. Erythrocytes prepared from sheep whole blood (Nippon Bio‐Test Laboratories, Asaka, Japan) were washed three times with PBS and resuspended in PBS at 1% (v/v). The erythrocyte suspension was added to a round‐bottomed 96‐well microtiter plate in 100 μL aliquots and incubated at room temperature for 4 h. The plate was then visually examined for erythrocyte precipitation (Shi et al. 1999).

### 2.12 Protease Activity Assay

*P. gingivalis* was cultured under anaerobic conditions, washed three times with PBS, and resuspended in 1 mL of PBS to achieve an optical density at 600 nm of 1.0. Bz‐l‐Arg‐pNA‐HCl (l‐BAPA: Peptide Institute, Osaka, Japan) was used as a substrate for the protease assay. l‐BAPA was dissolved in sterile distilled water to a final concentration of 1 mM to prepare the substrate solution. An equal volume of substrate solution was mixed with the bacteria and Tris‐HCl buffer (pH 8.0) and incubated at 37°C for 30 min to allow for the enzymatic reaction. The protease activity was measured by quantifying the concentration of free *p*‐nitroanilide at an absorbance of 405 nm (Sztukowska et al. 2004).

### 2.13 Immunofluorescent Staining

Macrophage‐like cells infected with *P. gingivalis* were fixed in PBS containing 4% PFA for 10 min and permeabilized with PBS containing 0.1% Triton X‐100 for 5 min. The cells were then incubated with a primary antibody, rabbit anti‐pSTAT1 (#7649, 1:500, Cell Signaling Technology, Danvers, MA, USA), for 1 h at 37°C, followed by incubation with a secondary antibody, Alexa Fluor 488‐conjugated alpaca anti‐rabbit IgG (1:1000, Thermo Fisher Scientific), for 1 h at 37°C in the dark. The cytoskeleton was stained simultaneously using Phalloidin‐iFluor 555 Reagent (1:1000, Abcam, Cambridge, UK) along with the secondary antibody. The stained cells were mounted on glass slides using Hard‐Set Mounting Medium with DAPI (Vector Laboratories, Newark, CA, USA) and analyzed using a confocal laser microscope (BZ‐X800, Keyence, Osaka, Japan).

### 2.14 Colony‐Forming Unit (CFU) Assay

Macrophage‐like cells infected with *P. gingivalis* were treated with 300 μg/mL gentamicin (Nacalai Tesque) and 200 μg/mL metronidazole (Nacalai Tesque) for 1 h at 37°C, 5% CO_2_, and 100% humidity to eliminate *P. gingivalis* on the cell membrane and in the medium. The cells were then washed twice with PBS and incubated with 1 mL of sterile distilled water for 30 min. Subsequently, the cells were plated onto Anero Columbia RS blood agar (Nippon Becton Dickinson) and incubated under anaerobic conditions until visible colonies appeared. The number of intracellular invading bacteria was determined by counting the colonies formed.

### 2.15 Statistical Analysis

Differences between two groups were analyzed using the Student's *t*‐test. One‐way ANOVA was used to compare three or more groups, followed by multiple comparison tests using the Tukey test. Prism software (GraphPad, La Jolla, CA, USA) was used for all data analysis, and a *p* value < 0.05 was considered statistically significant.

## 3 Results

### 3.1 *P. gingivalis* Attenuates IFN‐γ‐Inducible Gene Expression to Modulate Immune Responses

**Figure 1:**
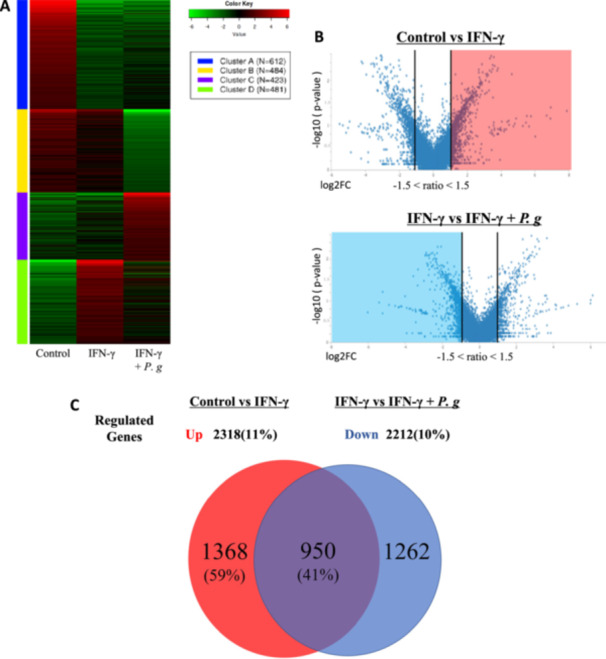
*P. gingivalis* attenuates IFN‐γ‐inducible gene expression to modulate immune responses. (A) Heatmap and clustering analysis depicting the varied expressions of the detected genes. (B and C) Data analysis based on the varied expressions of genes by RNA‐seq. (B) Visualization of variation ratios and statistical test quantities of variably expressed genes by a volcano plot. (C) A Venn diagram created to show the varied expressions of genes extracted from volcano plots.

To comprehensively analyze the gene expression patterns induced by *P. gingivalis* infection in IFN‐γ‐stimulated macrophages, RNA‐seq data were processed using iDEP, a web‐based tool for RNA‐seq analysis. K‐means clustering identified four distinct gene expression clusters, and their patterns were visualized using a heatmap (Khan 2012) (Figure 1A). Cluster D displayed a unique expression pattern: genes were upregulated in the IFN‐γ group but downregulated in the IFN‐γ + *P. gingivalis* group compared with controls. The enrichment analysis of Cluster D highlighted a significant prevalence of IFN‐related genes, along with genes involved in immune responses and inflammatory pathways.

Additionally, volcano plots were generated to identify differentially expressed genes in the control, IFN‐γ, and IFN‐γ + *P. gingivalis* groups (Figure 1B). Genes with |log2 fold change| ≥ 1.5 were defined as differentially expressed. When comparing the control group with the IFN‐γ group, 21,073 genes showed altered expressions, of which 2318 genes met the criterion for upregulation. Similarly, when comparing the IFN‐γ group with the IFN‐γ + *P. gingivalis* group, 22,120 genes displayed altered expressions, of which 2212 genes met the criterion for downregulation. In addition to these gene sets, 715 genes that were downregulated by IFN‐γ alone and 465 genes that were upregulated upon IFN‐γ + *P. gingivalis* treatment were also observed in the volcano plots, although these were not further analyzed in the present study.

A Venn diagram was created using the Draw Venn Diagram tool to illustrate overlaps between gene sets (Figure 1C). The analysis identified 950 overlapping genes between the 2318 genes upregulated by IFN‐γ stimulation and the 2212 genes downregulated by *P. gingivalis* infection. This indicated that approximately 41% of genes upregulated following IFN‐γ stimulation showed reduced transcript abundance after subsequent *P. gingivalis* infection, highlighting a significant immune modulation mechanism.

### 3.2 *P. gingivalis* Infection Reduces STAT1 Transcript Abundance by a Time‐ and Dose‐Dependent Mechanism Across Diverse Cell Types and Strains

**Figure 2:**
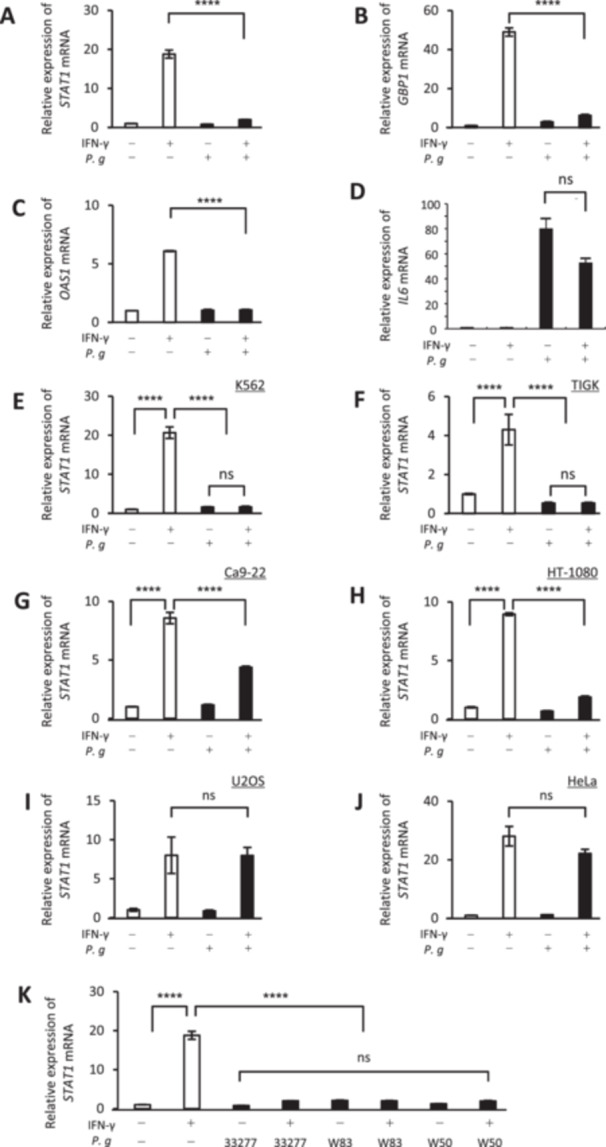
*P. gingivalis* infection reduces *STAT1* transcript abundance across diverse cell types and strains. (A–D) Verification of the suppression of inflammation‐related genes by *P. gingivalis*. Evaluation of gene expression levels of *STAT1* (A), *GBP1* (B), *OAS1* (C), and *IL6* (D) during *P. gingivalis* infection. Suppression of IFN signaling in different cell types, including K562 (E), TIGK (F), Ca9‐22 (G), HT‐1080 (H), U2OS (I), and HeLa (J) cell lines. (K) Suppression of IFN signaling by different strains of *P. gingivalis*. NS, not significant; ****p < 0.0001 (ANOVA with the Tukey test). All results are the mean ± standard deviation (SD) and are representative of three independent experiments.

Changes in gene expression in macrophage‐like cells stimulated with IFN‐γ and infected with *P. gingivalis* were analyzed by RT‐qPCR under the same conditions as the RNA‐seq study. Three target genes (*STAT1, GBP1*, and *OAS1*) were selected based on their ranking among the Top 30 IFN‐related genes with significantly suppressed expression in the IFN‐γ + *P. gingivalis* group (Table S4). RT‐qPCR confirmed that IFN‐γ stimulation increased the mRNA levels of these genes, whereas subsequent *P. gingivalis* infection reduced their transcript levels (Figure 2A–C). Notably, *STAT1*, which encodes a key transcription factor upstream of the IFN signaling pathway, was significantly suppressed, making it the focal target for subsequent experiments. However, the expression of *IL6*, representing an inflammatory cytokine gene that is not a canonical IFN‐stimulated gene (ISG), was not suppressed by *P. gingivalis* infection (Figure 2D).

The cell type and strain specificity of *P. gingivalis*‐mediated suppression of *STAT1* expression were evaluated next. Reduced *STAT1* mRNA levels were observed across multiple cell types, including K562 (hematopoietic), TIGK (oral epithelial), Ca9‐22 (gingival carcinoma), and HT‐1080 (fibrosarcoma) cells (Figure 2E–H). Conversely, no inhibitory effect was detected in U2OS (osteosarcoma) or HeLa (cervical cancer) cells (Figure 2I,J). Regarding *P. gingivalis* strains, this suppressive effect was consistently suppressed across ATCC 33277, W83, and W50, without any discernible strain‐specific differences (Figure 2K).

To assess whether the inhibitory effect of *P. gingivalis* infection on *STAT1* transcript abundance was time‐dependent, infections were conducted at a fixed MOI of 100, with time points of 6, 24, and 48 h. In all cases, *STAT1* mRNA levels were reduced, and the effect became more pronounced over time (Figure S1A). Similarly, a concentration‐dependent reduction in *STAT1* transcript abundance was observed when the infection time was fixed at 24 h, and the MOI was varied (10, 50, 100) (Figure S1B). These results indicate that *P. gingivalis*‐mediated suppression of *STAT1* expression intensified with prolonged infection and increased bacterial load.

### 3.3 Direct Contact and Intact Bacterial Membrane Proteins Are Essential for *P. gingivalis*‐Mediated Suppression of *STAT1* Gene Expression

**Figure 3:**
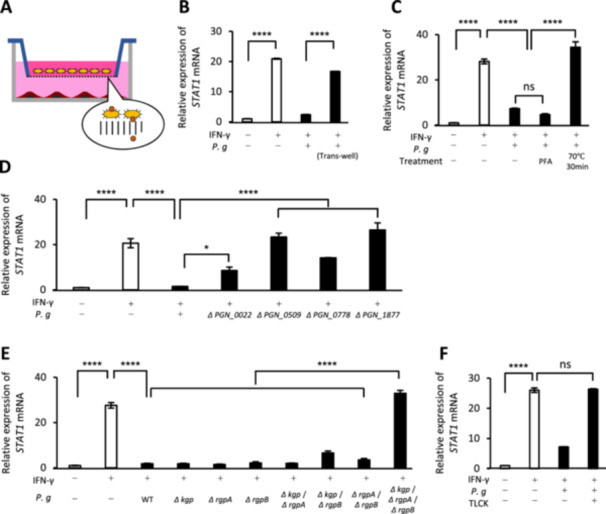
Type IX secretion system and gingipains are critical virulence factors for *P. gingivalis*‐mediated suppression of *STAT1* expression. (A) Schematic of the noncontact culture with Transwell inserts. (B) Evaluation of *STAT1* expression during *P. gingivalis* infection using Transwell inserts. (C) Effects of whole‐cell PFA fixation or heat treatment on the ability of *P. gingivalis* to suppress *STAT1* expression. (D and E) Exploration of bacterial virulence factors involved in the suppression of IFN signaling. (D) Evaluation of *STAT1* expression during infection with *PGN_0022*, *_0509*, *_0778*, and *_1877* knockout strains (T9SS). (E) Evaluation of *STAT1* expression during infection with single, double, and triple knockout strains of the gingipain genes *kgp, rgpA*, and *rgpB*. (F) Evaluation of *STAT1* expression during *P. gingivalis* infection in the presence of the gingipain inhibitor TLCK. NS, not significant; *p < 0.05; ****p < 0.0001 (ANOVA with the Tukey test). All results are the mean ± SD and are representative of three independent experiments.

To investigate whether the suppression of *STAT1* expression by *P. gingivalis* required direct contact with host cells or was mediated by secreted bacterial molecules, we used a noncontact co‐culture method with Transwells (pore size 0.4 μm), which separates bacteria and host cells. The use of Transwells did not lead to STAT1 suppression by *P. gingivalis* (Figure 3A,B).

Next, we examined whether intact bacterial surface‐associated proteins were required for suppression of *STAT1* expression. We treated *P. gingivalis* with 4% PFA in PBS, which fixes cells by crosslinking proteins and preserves overall surface architecture and membrane integrity. Under these conditions, suppression of *STAT1* expression was retained. Conversely, no suppression was observed when the bacteria were heat‐treated to denature all proteins (Figure 3C).

### 3.4 Type IX Secretion System and Gingipains Are Critical Virulence Factors for *P. gingivalis*‐Mediated Suppression of *STAT1* Transcript Abundance

The specificity of *P. gingivalis* interactions with its host arises from the diverse virulence factors it possesses, including LPS, fimbriae, capsule membrane, gingipain, and the outer membrane vesicles (OMVs; Xu et al. 2020; Aleksijević et al. 2022). Many of these virulence factors are known to induce inflammation by activating various signaling pathways in host cells.

To identify the virulence factors of *P. gingivalis* involved in the suppression of IFN signaling, we evaluated the effect of LPS (Gabarrini et al. 2020). *STAT1* gene expression was upregulated when LPS derived from *P. gingivalis* (LPS‐PG) was added following IFN‐γ stimulation (Figure S2A). Next, we generated mutants for the fimbriae‐associated genes *fimA*, *B*, *C*, *D*, and *mfa1–mfa5* (Figure S2B–D). Infection with these mutants reduced *STAT1* transcript levels to a similar extent as the WT strain (Figure S2E,F).

Mutant strains of genes encoding proteins associated with the outer membrane of *P. gingivalis* revealed that the genes *PGN_0022* (*porU*), *PGN_0509* (*porZ*), *PGN_0778* (*porT*), *PGN_1676* (*porK*), *PGN_1677* (*porN*), and *PGN_1877* (*porW*) led to the partial or complete loss of suppression of the inhibitory effect on *STAT1* expression (Figures 3D and S3A). These genes encode components of the Type IX Secretion System (T9SS), a protein secretion mechanism responsible for anchoring proteins to the bacterial outer membrane (Sato et al. 2010; Lasica et al. 2017). Based on these findings, we compiled a list of T9SS‐anchored genes and generated mutant strains for each (Table S5) (Glew et al. 2012; Veith et al. 2013, 2021). However, some mutants, such as *PGN_0693* and *PGN_0810*, had no effect on this suppressive activity (Figure S3B).

When the mutant strains that lost their inhibitory effect on *STAT1* were plated on blood agar, they produced white colonies (Figure S3C). This loss of pigmentation was previously observed in gingipain‐deficient strains (Okamoto et al. 1998; Shi et al. 1999), implicating T9SS in gingipain secretion (Nakayama 2015). To explore this connection further, we generated single, double, and triple knockout strains of the gingipain genes *kgp*, *rgpA*, and *rgpB* (Figure S4A–I). Notably, the triple knockout strain completely lost its ability to suppress *STAT1* expression, whereas the single and double knockout strains retained this ability, similar to the WT strain (Figure 3E). Additionally, the gingipain inhibitor TLCK abolished the inhibitory effect of *P. gingivalis* on *STAT1* expression (Figure 3F). Functional analyses of gingipain in the triple knockout strain confirmed the loss of gingipain activity, as evidenced by the absence of pigmentation on blood agar, reduced protease activity in l‐BAPA assays, and impaired erythrocyte agglutination (Figure S5A–C).

### 3.5 Gingipains Facilitate *P. gingivalis* Invasion and Inhibit pSTAT1 Nuclear Translocation During IFN‐γ Stimulation

**Figure 4:**
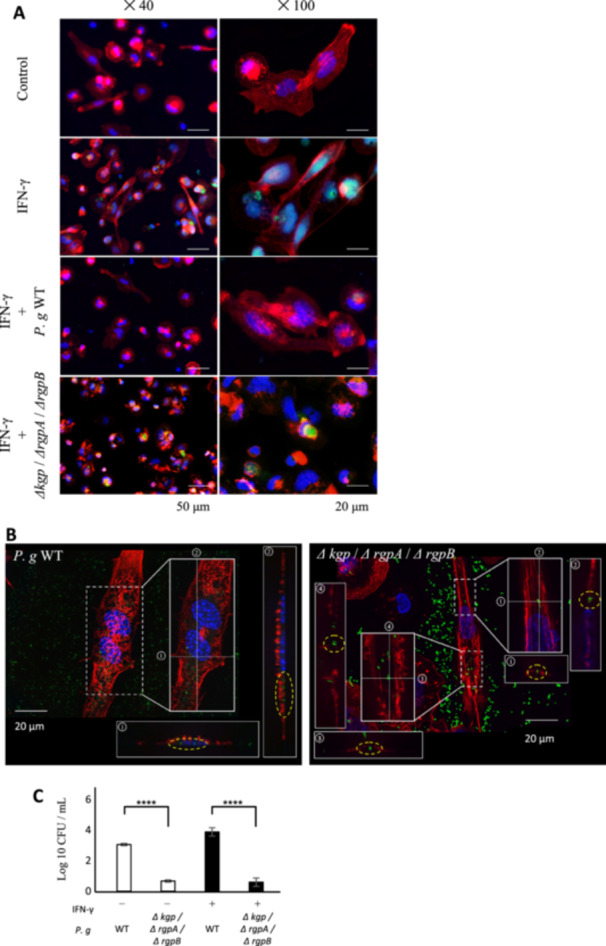
Gingipains facilitate *P. gingivalis* invasion and inhibit STAT1 nuclear translocation during IFN‐γ stimulation. (A) Evaluation of pSTAT1 localization during infection with *P. gingivalis* WT and gingipain gene triple knockout strains using immunofluorescence staining (nuclei: blue, actin: red, pSTAT1: green). (B and C) Dynamics of *P. gingivalis* during the suppression of IFN signaling. (B) 3D analysis of immunofluorescence staining during infection with *P. gingivalis* WT and a gingipain gene triple knockout strain (nuclei: blue, actin: red, *P. gingivalis*: green). (C) Number of *P. gingivalis* WT and gingipain gene triple knockout strains that invaded cells and survived, measured by CFU assay. NS, not significant; ****p < 0.0001 (ANOVA with the Tukey test).

To evaluate the activation and intracellular behavior of STAT1 upon IFN‐γ stimulation and *P. gingivalis* infection, we performed immunofluorescence staining using an anti‐phosphorylated STAT1 (pSTAT1) antibody. The nuclear translocation of pSTAT1, which was observed in IFN‐γ‐stimulated cells, was abolished upon infection with *P. gingivalis* WT. In contrast, no inhibition of pSTAT1 nuclear translocation waas observed in the triple knockout strain lacking all three gingipains (Figure 4A). Subsequently, *P. gingivalis* WT or the triple knockout strain was stained with FITC, and the dynamics of bacterial invasion during cell infection were examined using immunofluorescence staining. Three‐dimensional reconstruction and cross‐sectional views of the stained cells revealed that *P. gingivalis* WT invaded the cells, whereas the triple knockout strain had significantly reduced invasion, with most bacteria remaining near the cell membrane (Figure 4B).

To determine whether the intracellular presence of *P. gingivalis* was related to phagocytosis or its invasive capacity, we quantified the number of intracellular invasions of *P. gingivalis* WT and the triple knockout strain lacking gingipain activity using the colony‐forming unit (CFU) assay. The results demonstrated that, regardless of IFN‐γ‐stimulated activation, the triple knockout strain had a significantly reduced intracellular invasion capability compared with *P. gingivalis* WT (Figure 4C).

## 4 Discussion

Our study revealed the specific regulation of the IFN‐γ signaling pathway through the comprehensive analysis of gene expression patterns in IFN‐γ–activated macrophages infected with *P. gingivalis*. IFN‐γ, an activator of macrophages, is critical for host defense and inflammatory responses against pathogens (Hu and Ivashkiv 2009). Downstream of IFN‐γ receptor signaling, STAT1 activation promotes the transcription of ISGs such as *IRF1*, *OAS1*, and *GBP1*, thereby activating the host immune response (Stark and Darnell 2012). Previous studies have shown elevated IFN‐γ levels in gingival crevicular fluid and periodontal tissues during periodontitis progression, which contribute to macrophage activation and tissue inflammation (Dutzan et al. 2009; Fiorillo et al. 2018). By interfering with IFN‐γ signaling even at low abundance, *P. gingivalis* may effectively dampen host immune responses and promote its persistence within the periodontal niche, thereby facilitating dysbiosis and disease progression. Among these ISGs, STAT1 serves as a central transcription factor upstream of the IFN‐γ signaling pathway (Aaronson and Horvath 2002). However, *P. gingivalis* infection strongly reduces *STAT1* transcript levels, resulting in negative regulation of IFN‐γ signaling.

Recent studies have demonstrated that *P. gingivalis* can modulate interferon‐related pathways in various contexts. For instance, Kgp‐mediated inhibition of IFN pathways was shown to promote viral infection (Dobosz et al. 2025), while OMVs suppressed innate immune surveillance in oral epithelial cells (Q. Chen et al. 2025). In contrast, our study focused specifically on macrophage activation under IFN‐γ stimulation and identified a direct, contact‐dependent mechanism involving the T9SS–gingipain system that suppresses *STAT1* expression. This provides novel mechanistic insight into how *P. gingivalis* evades IFN‐mediated macrophage immunity, distinct from previously reported virally linked or tumorigenic models.

*P. gingivalis* is a Gram‐negative anaerobic bacterium that causes severe or chronic periodontal disease (Lamont and Jenkinson 1998). Furthermore, the immunosuppressive mechanisms of this bacterium affect immunocompetent cells as well as oral epithelial cells. Interestingly, suppression of *STAT1* expression was not observed in human osteosarcoma or cervical cancer cell lines, suggesting that this phenotype may depend on host cell‐specific gene expression programs. *P. gingivalis* strains have also been classified according to their fimbrial genotypes and virulence‐associated phenotypes. ATCC 33277 is generally regarded as a less‐virulent strain, whereas W83 is widely considered more virulent in experimental models; W50 has likewise been used as a virulent laboratory strain in studies of *P. gingivalis* pathogenicity (Marsh et al. 1994; T. Chen et al. 2004; Naito et al. 2008). With regard to fimbriae, ATCC 33277 carries type I FimA, whereas W83 and W50 are associated with type IV FimA (Enersen et al. 2013). In the present study, suppression of *STAT1* expression was observed in ATCC 33277, W83, and W50, indicating that this IFN‐γ‐inhibitory phenotype is conserved across the strains tested. Consistent with this observation, mutants lacking major fimbriae‐associated genes (*fimA*–*D* and *mfa1*–*5*) retained the ability to suppress *STAT1* expression, suggesting that fimbrial components are not essential for this phenotype in our experimental system.

Pathogenic factors of *P. gingivalis* can be classified into two categories: localized factors anchored on the bacterial outer membrane and secretory factors released extracellularly (Mohanty et al. 2019; Gabarrini et al. 2020). Transwell isolation experiments, together with whole‐cell PFA and heat treatments of *P. gingivalis*, demonstrated that surface‐localized molecules are essential for the inhibition of IFN signaling. Furthermore, PFA‐treated *P. gingivalis* retained the ability to suppress *STAT1* transcript abundance, suggesting that this immunosuppressive function can persist in nonviable bacteria as long as relevant surface‐associated protein structures remain sufficiently preserved. When macrophages phagocytose pathogens, degraded protein fragments are presented on the cell surface, thereby contributing to antigen presentation and immune activation (Ackerman and Cresswell 2004). However, it was reported that macrophages inhibit the production of inflammatory mediators when phagocytosing apoptotic cells (Fadok et al. 1998). Therefore, immune cells may still recognize foreign proteins after phagocytosis and activate specific signaling pathways. Although it has been shown that molecules present on the outer membrane of *P. gingivalis* are important for the suppression of IFN signaling, further verification is required as to whether the virulence factors exert their effects on the host cell membrane or intracellularly.

While gingipains are known to be major cargo of *P. gingivalis* OMVs (Okamura et al. 2021), and these vesicles can deliver gingipains into the cytosol of host cells (Nonaka et al. 2024), our Transwell assay results indicate that OMV‐mediated delivery alone may not suffice to suppress *STAT1* expression in the absence of direct bacterial contact. This observation suggests that the concentration or functional integrity of OMVs under our experimental conditions might have been insufficient, or that additional mechanisms, such as intracellular trafficking and signal interference following bacterial invasion, are required for effective suppression of IFN signaling.

Typical virulence factors include LPS, fimbriae, and capsules; however, these factors did not suppress *STAT1* expression. Approximately 124 proteins localized on the *P. gingivalis* outer membrane have been reported (Gabarrini et al. 2020). Among the membrane proteins, T9SS‐associated proteins influenced this phenotype. These proteins, including those with a specific C‐terminal domain, function by anchoring to the bacterial membrane or interacting with other proteins. Currently, 35 proteins related to T9SS and anchored on the bacterial membrane have been reported (Table S5) (Lasica et al. 2017; Veith et al. 2013; Gorasia et al. 2020). In our study, we created mutant strains of all 35 proteins and verified that not all of them were involved in the suppression of IFN signaling. Furthermore, the degree of suppression varies. In interpreting the effects of individual T9SS substrate mutants, it should also be considered that the apparent impact on host signaling may depend on the relative abundance and condition‐dependent expression/processing of each T9SS cargo protein. The T9SS is involved in the secretion of gingipains, and some strains lacking T9SS‐related genes had impaired gingipain secretion (Veillard et al. 2019). Therefore, the differences observed in the strains lacking T9SS‐related genes in this study may be related to impaired gingipain secretion.

Previous studies have demonstrated that gingipains can directly degrade IFN‐γ, thereby reducing its bioavailability and impairing downstream immune activation (Yun et al. 1999). This proteolytic inactivation represents one of the earliest described mechanisms by which *P. gingivalis* interferes with interferon‐mediated host defense. However, our results reveal an additional, distinct level of regulation—suppression of *STAT1* expression within macrophages—indicating that *P. gingivalis* modulates the IFN‐γ pathway not only by extracellular cytokine degradation but also by intracellular transcriptional suppression. Together, these mechanisms suggest a multilayered immune evasion strategy that operates both outside and inside host cells.

There are three types of gingipains, Kgp, RgpA, and RgpB, which have protease activity and interact in a complex manner with other virulence factors present on the bacterial surface (Guo et al. 2010). Kgp and RgpA contain domains involved in proteolysis as well as erythrocyte aggregation and adhesion, whereas RgpB mainly comprises a protease domain (O'Brien‐Simpson et al. 2001; Kamaguchi et al. 2003). In the *kgp* and *rgpA*‐deficient strains generated in this study, single or double deletion did not alter the inhibition of IFN signaling, indicating that the domains involved in erythrocyte aggregation and adhesion in the gingipains were not essential for this phenotype. In a triple deletion strain of *kgp*, *rgpA*, and *rgpB*, the inhibition of IFN signaling was lost. Furthermore, similar results were obtained when the protease function of *P. gingivalis* was inhibited using TLCK. Together, these results indicate the importance of the gingipain‐dependent proteolysis in the suppression of IFN signaling by *P. gingivalis*. It should be noted, however, that gingipain deficiency can have broader consequences beyond loss of gingipain activity itself, because multiple *P. gingivalis* proteins are processed and matured in a gingipain‐dependent manner. Therefore, the phenotype observed in the *ΔkgpΔrgpAΔrgpB* strain may also reflect indirect effects on other surface/cargo proteins, including T9SS substrates. Moreover, because TLCK can inhibit other proteases, including serine proteases, the TLCK‐based inhibition data should be interpreted cautiously.

As previously observed in epithelial cells (T. Chen et al. 2001), gingipain‐deficient strains also showed reduced adhesion and intracellular invasion rates in macrophage‐like cell lines. Therefore, the STAT1 inhibitory effect of *P. gingivalis* is thought to be caused by bacterial invasion into cells, indicating that gingipain protease is an intracellular invasive factor as well as a host immunosuppressant.

## 5 Conclusion

The immunosuppressive mechanism of *P. gingivalis* is mediated by the protease activity of gingipains, which also promotes bacterial invasion into host cells. These findings suggest that suppression of interferon signaling is a downstream consequence of intracellular infection. Future studies should aim to identify the host factors and signaling pathways targeted by this process.

## Author Contributions

**Shotaro Abe:** conceptualization, methodology, investigation, writing – original draft, writing – review and editing, funding acquisition. **Jun Ohshima:** conceptualization, methodology, investigation, writing – original draft, writing – review and editing, funding acquisition, supervision. **Masayoshi Morita:** investigation, funding acquisition. **Nobutake Tanaka:** investigation. **Mikako Hayashi:** investigation, writing – review and editing, funding acquisition, supervision. All authors approved the final version to be published and agreed to be accountable for all aspects of this work.

## Ethics Statement

The authors have nothing to report.

## Conflicts of Interest

The authors declare no conflicts of interest.

## Supporting information

Supporting File 1

## Data Availability

The data supporting the findings of this study are available at Figshare https://doi.org/10.6084/m9.figshare.28553744. RNA‐seq data generated in this paper have been deposited into the DNA Data Bank of Japan (DDBJ) sequence read archive (https://www.ddbj.nig.ac.jp/bioproject/index-e.html) and are publicly available as of the date of publication (BioProject accession number PRJDB19816: https://www.ncbi.nlm.nih.gov/bioproject/?term=PRJDB19816). Supplemental data for this article can be accessed at https://doi.org/10.6084/m9.figshare.28564397.

## References

[mbo370290-bib-0001] Aaronson, D. S. , and C. M. Horvath . 2002. “A Road Map for Those Who Don't Know JAK‐STAT.” Science 296, no. 5573: 1653–1655. 10.1126/science.1071545. 12040185

[mbo370290-bib-0002] Abusleme, L. , A. K. Dupuy , N. Dutzan , et al. 2013. “The Subgingival Microbiome in Health and Periodontitis and Its Relationship With Community Biomass and Inflammation.” ISME Journal 7, no. 5: 1016–1025. 10.1038/ismej.2012.174. 23303375 PMC3635234

[mbo370290-bib-0003] Ackerman, A. L. , and P. Cresswell . 2004. “Cellular Mechanisms Governing Cross‐Presentation of Exogenous Antigens.” Nature Immunology 5, no. 7: 678–684. 10.1038/ni1082. 15224093

[mbo370290-bib-0004] Aleksijević, L. H. , M. Aleksijević , I. Škrlec , M. Šram , M. Šram , and J. Talapko . 2022. “ *Porphyromonas gingivalis* Virulence Factors and Clinical Significance in Periodontal Disease and Coronary Artery Diseases.” Pathogens 11, no. 10: 1173. 10.3390/pathogens11101173. 36297228 PMC9609396

[mbo370290-bib-0005] Chen, T. , Y. Hosogi , K. Nishikawa , et al. 2004. “Comparative Whole‐Genome Analysis of Virulent and Avirulent Strains of *Porphyromonas gingivalis* .” Journal of Bacteriology 186, no. 16: 5473–5479. 10.1128/JB.186.16.5473-5479.2004. 15292149 PMC490943

[mbo370290-bib-0006] Chen, T. , K. Nakayama , L. Belliveau , and M. J. Duncan . 2001. “ *Porphyromonas gingivalis* Gingipains and Adhesion to Epithelial Cells.” Infection and Immunity 69, no. 5: 3048–3056. 10.1128/IAI.69.5.3048-3056.2001. 11292723 PMC98259

[mbo370290-bib-0007] Chen, Q. , X. Pang , K. Liu , et al. 2025. “ *Porphyromonas gingivalis* Outer Membrane Vesicles Promote Oral Tumorigenesis Through Suppressing Innate Immune Surveillance.” Microbiological Research 301: 128296. 10.1016/j.micres.2025.128296. 40774042

[mbo370290-bib-0008] Cremer, T. J. , D. H. Ravneberg , C. D. Clay , et al. 2009. “MiR‐155 Induction by *F. novicida* but Not the Virulent *F. tularensis* Results in SHIP Down‐Regulation and Enhanced Pro‐Inflammatory Cytokine Response.” PLoS One 4, no. 12: e8508. 10.1371/journal.pone.0008508. 20041145 PMC2794384

[mbo370290-bib-0009] Deo, P. , and R. Deshmukh . 2019. “Oral Microbiome: Unveiling the Fundamentals.” Journal of Oral and Maxillofacial Pathology 23, no. 1: 122. 10.4103/jomfp.JOMFP_304_18. PMC6503789 31110428

[mbo370290-bib-0010] Dickinson, B. C. , C. E. Moffatt , D. Hagerty , et al. 2011. “Interaction of Oral Bacteria With Gingival Epithelial Cell Multilayers.” Molecular Oral Microbiology 26, no. 3: 210–220. 10.1111/j.2041-1014.2011.00609.x. 21545698 PMC3248246

[mbo370290-bib-0011] Dobosz, E. , A. Golda , M. Kanoza , et al. 2025. “Lys‐Specific Gingipain (Kgp) of *P. gingivalis* Promotes Viral Infection by Disabling the Interferon Pathway.” mBio 16, no. 10: e00298‐25. 10.1128/mbio.00298-25. 40874777 PMC12506115

[mbo370290-bib-0012] Dominy, S. S. , C. Lynch , F. Ermini , et al. 2019. “ *Porphyromonas gingivalis* in Alzheimer's Disease Brains: Evidence for Disease Causation and Treatment With Small‐Molecule Inhibitors.” Science Advances 5, no. 1: eaau3333. 10.1126/sciadv.aau3333. 30746447 PMC6357742

[mbo370290-bib-0013] Dutzan, N. , R. Vernal , M. Hernandez , et al. 2009. “Levels of Interferon‐Gamma and Transcription Factor T‐Bet in Progressive Periodontal Lesions in Patients With Chronic Periodontitis.” Journal of Periodontology 80, no. 2: 290–296. 10.1902/jop.2009.080287. 19186970

[mbo370290-bib-0014] Enersen, M. , K. Nakano , and A. Amano . 2013. “ *Porphyromonas gingivalis* Fimbriae.” Journal of Oral Microbiology 5, no. 1: 20265. 10.3402/jom.v5i0.20265. PMC3647041 23667717

[mbo370290-bib-0015] Fadok, V. A. , D. L. Bratton , A. Konowal , P. W. Freed , J. Y. Westcott , and P. M. Henson . 1998. “Macrophages That Have Ingested Apoptotic Cells In Vitro Inhibit Proinflammatory Cytokine Production Through Autocrine/Paracrine Mechanisms Involving TGF‐Beta, PGE2, and PAF.” Journal of Clinical Investigation 101, no. 4: 890–898. 10.1172/JCI1112. 9466984 PMC508637

[mbo370290-bib-0016] Fiorillo, L. , G. Cervino , A. S. Herford , et al. 2018. “Interferon Crevicular Fluid Profile and Correlation With Periodontal Disease and Wound Healing: A Systemic Review of Recent Data.” International Journal of Molecular Sciences 19, no. 7: 1908. 10.3390/ijms19071908. 29966238 PMC6073775

[mbo370290-bib-0017] Gabarrini, G. , S. Grasso , A. J. Van Winkelhoff , and J. M. Van Dijl . 2020. “Gingimaps: Protein Localization in the Oral Pathogen *Porphyromonas gingivalis* .” Microbiology and Molecular Biology Reviews 84, no. 1: e00032‐19. 10.1128/MMBR.00032-19. 31896547 PMC6941882

[mbo370290-bib-0018] Ge, S. X. , E. W. Son , and R. Yao . 2018. “iDEP: An Integrated Web Application for Differential Expression and Pathway Analysis of RNA‐Seq Data.” BMC Bioinformatics 19, no. 1: 534. 10.1186/s12859-018-2486-6. 30567491 PMC6299935

[mbo370290-bib-0019] Glew, M. D. , P. D. Veith , B. Peng , et al. 2012. “PG0026 Is the C‐Terminal Signal Peptidase of a Novel Secretion System of *Porphyromonas gingivalis* .” Journal of Biological Chemistry 287, no. 29: 24605–24617. 10.1074/jbc.M112.369223. 22593568 PMC3397888

[mbo370290-bib-0020] Gorasia, D. G. , P. D. Veith , and E. C. Reynolds . 2020. “The Type IX Secretion System: Advances in Structure, Function and Organisation.” Microorganisms 8, no. 8: 1173. 10.3390/microorganisms8081173. 32752268 PMC7463736

[mbo370290-bib-0021] Guo, Y. , K.‐A. Nguyen , and J. Potempa . 2010. “Dichotomy of Gingipains Action as Virulence Factors: From Cleaving Substrates With the Precision of a Surgeon's Knife to a Meat Chopper‐Like Brutal Degradation of Proteins: Gingipains as Main Virulence Factors of *P. gingivalis* .” Periodontology 2000 54, no. 1: 15–44. 10.1111/j.1600-0757.2010.00377.x. 20712631 PMC2924770

[mbo370290-bib-0022] Hajishengallis, G. 2014. “Immunomicrobial Pathogenesis of Periodontitis: Keystones, Pathobionts, and Host Response.” Trends in Immunology 35, no. 1: 3–11. 10.1016/j.it.2013.09.001. 24269668 PMC3947349

[mbo370290-bib-0023] Hajishengallis, G. 2015. “Periodontitis: From Microbial Immune Subversion to Systemic Inflammation.” Nature Reviews Immunology 15, no. 1: 30–44. 10.1038/nri3785. PMC4276050 25534621

[mbo370290-bib-0024] Hajishengallis, G. , and T. Chavakis . 2021. “Local and Systemic Mechanisms Linking Periodontal Disease and Inflammatory Comorbidities.” Nature Reviews Immunology 21, no. 7: 426–440. 10.1038/s41577-020-00488-6. PMC7841384 33510490

[mbo370290-bib-0025] Hajishengallis, G. , S. Liang , M. A. Payne , et al. 2011. “Low‐Abundance Biofilm Species Orchestrates Inflammatory Periodontal Disease Through the Commensal Microbiota and Complement.” Cell Host & Microbe 10, no. 5: 497–506. 10.1016/j.chom.2011.10.006. 22036469 PMC3221781

[mbo370290-bib-0026] Hu, X. , and L. B. Ivashkiv . 2009. “Cross‐Regulation of Signaling Pathways by Interferon‐γ: Implications for Immune Responses and Autoimmune Diseases.” Immunity 31, no. 4: 539–550. 10.1016/j.immuni.2009.09.002. 19833085 PMC2774226

[mbo370290-bib-0027] Hulsen, T. , J. De Vlieg , and W. Alkema . 2008. “BioVenn – A Web Application for the Comparison and Visualization of Biological Lists Using Area‐Proportional Venn Diagrams.” BMC Genomics 9, no. 1: 488. 10.1186/1471-2164-9-488. 18925949 PMC2584113

[mbo370290-bib-0028] Jauregui, C. E. , Q. Wang , C. J. Wright , H. Takeuchi , S. M. Uriarte , and R. J. Lamont . 2013. “Suppression of T‐Cell Chemokines by *Porphyromonas gingivalis* .” Infection and Immunity 81, no. 7: 2288–2295. 10.1128/IAI.00264-13. 23589576 PMC3697598

[mbo370290-bib-0029] Jung, Y.‐J. , D. P. Miller , J. D. Perpich , et al. 2019. “ *Porphyromonas gingivalis* Tyrosine Phosphatase Php1 Promotes Community Development and Pathogenicity.” mBio 10, no. 5: e02004‐19. 10.1128/mBio.02004-19. 31551334 PMC6759763

[mbo370290-bib-0030] Kamaguchi, A. , T. Ohyama , E. Sakai , et al. 2003. “Adhesins Encoded by the Gingipain Genes of *Porphyromonas gingivalis* Are Responsible for Co‐Aggregation With *Prevotella intermedia* .” Microbiology 149, no. 5: 1257–1264. 10.1099/mic.0.25997-0. 12724387

[mbo370290-bib-0031] Khan, F. 2012. “An Initial Seed Selection Algorithm for K‐Means Clustering of Georeferenced Data to Improve Replicability of Cluster Assignments for Mapping Application.” Applied Soft Computing 12, no. 11: 3698–3700. 10.1016/j.asoc.2012.07.021.

[mbo370290-bib-0032] Kolliniati, O. , E. Ieronymaki , E. Vergadi , and C. Tsatsanis . 2022. “Metabolic Regulation of Macrophage Activation.” Journal of Innate Immunity 14, no. 1: 51–68. 10.1159/000516780. 34247159 PMC8787535

[mbo370290-bib-0033] Lamont, R. J. , and H. F. Jenkinson . 1998. “Life Below the Gum Line: Pathogenic Mechanisms of *Porphyromonas gingivalis* .” Microbiology and Molecular Biology Reviews 62, no. 4: 1244–1263. 10.1128/MMBR.62.4.1244-1263.1998. 9841671 PMC98945

[mbo370290-bib-0034] Lasica, A. M. , M. Ksiazek , M. Madej , and J. Potempa . 2017. “The Type IX Secretion System (T9SS): Highlights and Recent Insights Into Its Structure and Function.” Frontiers in Cellular and Infection Microbiology 7: 215. 10.3389/fcimb.2017.00215. 28603700 PMC5445135

[mbo370290-bib-0035] Maekawa, T. , J. L. Krauss , T. Abe , et al. 2014. “ *Porphyromonas gingivalis* Manipulates Complement and TLR Signaling to Uncouple Bacterial Clearance From Inflammation and Promote Dysbiosis.” Cell Host & Microbe 15, no. 6: 768–778. 10.1016/j.chom.2014.05.012. 24922578 PMC4071223

[mbo370290-bib-0036] Marsh, P. D. , A. S. McDermid , A. S. McKee , and A. Baskerville . 1994. “The Effect of Growth Rate and Haemin on the Virulence and Proteolytic Activity of *Porphyromonas gingivalis* W50.” Microbiology 140, no. 4: 861–865. 10.1099/00221287-140-4-861. 8012602

[mbo370290-bib-0037] Mohanty, R. , S. J. Asopa , M. Joseph , et al. 2019. “Red Complex: Polymicrobial Conglomerate in Oral Flora: A Review.” Journal of Family Medicine and Primary Care 8, no. 11: 3480–3486. 10.4103/jfmpc.jfmpc_759_19. PMC6881954 31803640

[mbo370290-bib-0038] Naito, M. , H. Hirakawa , A. Yamashita , et al. 2008. “Determination of the Genome Sequence of *Porphyromonas gingivalis* Strain ATCC 33277 and Genomic Comparison With Strain W83 Revealed Extensive Genome Rearrangements in *P. gingivalis* .” DNA Research 15, no. 4: 215–225. 10.1093/dnares/dsn013. 18524787 PMC2575886

[mbo370290-bib-0039] Nakayama, K. 2015. “ *Porphyromonas gingivalis* and Related Bacteria: From Colonial Pigmentation to the Type IX Secretion System and Gliding Motility.” Journal of Periodontal Research 50, no. 1: 1–8. 10.1111/jre.12255. 25546073 PMC4674972

[mbo370290-bib-0040] Nonaka, S. , R. Okamoto , Y. Katsuta , S. Kanetsuki , and H. Nakanishi . 2024. “Gingipain‐Carrying Outer Membrane Vesicles From *Porphyromonas gingivalis* Cause Barrier Dysfunction of Caco‐2 Cells by Releasing Gingipain Into the Cytosol.” Biochemical and Biophysical Research Communications 707: 149783. 10.1016/j.bbrc.2024.149783. 38493746

[mbo370290-bib-0041] O'Brien‐Simpson, N. M. , R. A. Paolini , B. Hoffmann , N. Slakeski , S. G. Dashper , and E. C. Reynolds . 2001. “Role of RgpA, RgpB, and Kgp Proteinases in Virulence of *Porphyromonas gingivalis* W50 in a Murine Lesion Model.” Infection and Immunity 69, no. 12: 7527–7534. 10.1128/IAI.69.12.7527-7534.2001. 11705929 PMC98843

[mbo370290-bib-0042] Okamoto, K. , K. Nakayama , T. Kadowaki , N. Abe , D. B. Ratnayake , and K. Yamamoto . 1998. “Involvement of a Lysine‐Specific Cysteine Proteinase in Hemoglobin Adsorption and Heme Accumulation by *Porphyromonas gingivalis* .” Journal of Biological Chemistry 273, no. 33: 21225–21231. 10.1074/jbc.273.33.21225. 9694880

[mbo370290-bib-0043] Okamura, H. , K. Hirota , K. Yoshida , et al. 2021. “Outer Membrane Vesicles of *Porphyromonas gingivalis*: Novel Communication Tool and Strategy.” Japanese Dental Science Review 57: 138–146. 10.1016/j.jdsr.2021.07.003. 34484474 PMC8399048

[mbo370290-bib-0044] Paster, B. J. , S. K. Boches , J. L. Galvin , et al. 2001. “Bacterial Diversity in Human Subgingival Plaque.” Journal of Bacteriology 183, no. 12: 3770–3783. 10.1128/JB.183.12.3770-3783.2001. 11371542 PMC95255

[mbo370290-bib-0045] Prieto, C. , and D. Barrios . 2020. “RaNA‐Seq: Interactive RNA‐Seq Analysis From FASTQ Files to Functional Analysis.” Bioinformatics 36, no. 6: 1955–1956. 10.1093/bioinformatics/btz854. 31730197

[mbo370290-bib-0046] Pussinen, P. J. , P. Jousilahti , G. Alfthan , T. Palosuo , S. Asikainen , and V. Salomaa . 2003. “Antibodies to Periodontal Pathogens Are Associated With Coronary Heart Disease.” Arteriosclerosis, Thrombosis, and Vascular Biology 23, no. 7: 1250–1254. 10.1161/01.ATV.0000072969.71452.87. 12714435

[mbo370290-bib-0047] Rodriguez‐Hernandez, C. J. , K. J. Sokoloski , K. S. Stocke , et al. 2021. “Microbiome‐Mediated Incapacitation of Interferon Lambda Production in the Oral Mucosa.” Proceedings of the National Academy of Sciences 118, no. 51: e2105170118. 10.1073/pnas.2105170118. PMC8713781 34921113

[mbo370290-bib-0048] Sato, K. , M. Naito , H. Yukitake , et al. 2010. “A Protein Secretion System Linked to Bacteroidete Gliding Motility and Pathogenesis.” Proceedings of the National Academy of Sciences 107, no. 1: 276–281. 10.1073/pnas.0912010107. PMC2806738 19966289

[mbo370290-bib-0049] Schoenborn, J. R. , and C. B. Wilson . 2007. “Regulation of Interferon‐γ During Innate and Adaptive Immune Responses.” In *Advances in Immunology*, 41–101. Elsevier. 10.1016/S0065-2776(07)96002-2. 17981204

[mbo370290-bib-0050] Shi, Y. , D. B. Ratnayake , K. Okamoto , N. Abe , K. Yamamoto , and K. Nakayama . 1999. “Genetic Analyses of Proteolysis, Hemoglobin Binding, and Hemagglutination of *Porphyromonas gingivalis* .” Journal of Biological Chemistry 274, no. 25: 17955–17960. 10.1074/jbc.274.25.17955. 10364243

[mbo370290-bib-0051] Stark, G. R. , and J. E. Darnell . 2012. “The JAK‐STAT Pathway at Twenty.” Immunity 36, no. 4: 503–514. 10.1016/j.immuni.2012.03.013. 22520844 PMC3909993

[mbo370290-bib-0052] Suzuki, T. A. , and R. E. Ley . 2020. “The Role of the Microbiota in Human Genetic Adaptation.” Science 370, no. 6521: eaaz6827. 10.1126/science.aaz6827. 33273073

[mbo370290-bib-0053] Sztukowska, M. , A. Sroka , M. Bugno , et al. 2004. “The C‐Terminal Domains of the Gingipain K Polyprotein Are Necessary for Assembly of the Active Enzyme and Expression of Associated Activities.” Molecular Microbiology 54, no. 5: 1393–1408. 10.1111/j.1365-2958.2004.04357.x. 15554977

[mbo370290-bib-0054] Tan, Q. , X. Ma , B. Yang , et al. 2022. “Periodontitis Pathogen *Porphyromonas gingivalis* Promotes Pancreatic Tumorigenesis via Neutrophil Elastase From Tumor‐Associated Neutrophils.” Gut Microbes 14, no. 1: 2073785. 10.1080/19490976.2022.2073785. 35549648 PMC9116393

[mbo370290-bib-0055] Thakur, A. , H. Mikkelsen , and G. Jungersen . 2019. “Intracellular Pathogens: Host Immunity and Microbial Persistence Strategies.” Journal of Immunology Research 2019: 1–24. 10.1155/2019/1356540. PMC6487120 31111075

[mbo370290-bib-0056] Veillard, F. , M. Sztukowska , Z. Nowakowska , et al. 2019. “Proteolytic Processing and Activation of Gingipain Zymogens Secreted by T9SS of *Porphyromonas gingivalis* .” Biochimie 166: 161–172. 10.1016/j.biochi.2019.06.010. 31212040 PMC6815250

[mbo370290-bib-0057] Veith, P. D. , D. G. Gorasia , and E. C. Reynolds . 2021. “Towards Defining the Outer Membrane Proteome of *Porphyromonas gingivalis* .” Molecular Oral Microbiology 36, no. 1: 25–36. 10.1111/omi.12320. 33124778

[mbo370290-bib-0058] Veith, P. D. , N. A. Nor Muhammad , S. G. Dashper , et al. 2013. “Protein Substrates of a Novel Secretion System Are Numerous in the Bacteroidetes Phylum and Have in Common a Cleavable C‐Terminal Secretion Signal, Extensive Post‐Translational Modification, and Cell‐Surface Attachment.” Journal of Proteome Research 12, no. 10: 4449–4461. 10.1021/pr400487b. 24007199

[mbo370290-bib-0059] Wang, X. , Y. Jia , L. Wen , et al. 2021. “ *Porphyromonas gingivalis* Promotes Colorectal Carcinoma by Activating the Hematopoietic *NLRP3* Inflammasome.” Cancer Research 81, no. 10: 2745–2759. 10.1158/0008-5472.CAN-20-3827. 34003774

[mbo370290-bib-0060] Wang, M. , J. L. Krauss , H. Domon , et al. 2010. “Microbial Hijacking of Complement–Toll‐Like Receptor Crosstalk.” Science Signaling 3, no. 109: ra11. 10.1126/scisignal.2000697. 20159852 PMC2824906

[mbo370290-bib-0061] Xu, W. , W. Zhou , H. Wang , and S. Liang . 2020. “Roles of *Porphyromonas gingivalis* and Its Virulence Factors in Periodontitis.” In Advances in Protein Chemistry and Structural Biology, 45–84. Elsevier. 10.1016/bs.apcsb.2019.12.001. PMC8204362 32085888

[mbo370290-bib-0062] Yun, P. L. W. , A. A. DeCarlo , and N. Hunter . 1999. “Modulation of Major Histocompatibility Complex Protein Expression by Human Gamma Interferon Mediated by Cysteine Proteinase‐Adhesin Polyproteins of *Porphyromonas gingivalis* .” Infection and Immunity 67, no. 6: 2986–2995. 10.1128/IAI.67.6.2986-2995.1999. 10338509 PMC96610

