# Supplementary material for: Porphyromonas gingivalis Suppresses Interferon‐Gamma Signaling in Macrophages Through a Contact‐Dependent, Gingipain‐Mediated Mechanism
Source: Microbiologyopen. 2026 Apr 12;15(2):e70290. doi: 10.1002/mbo3.70290 (PMC13070879; doi:10.1002/mbo3.70290)
Supplement: Supplementary file 1 — Supporting File 1 [file MBO3-15-e70290-s001.pdf]

Supplemental information

Porphyromonas gingivalis suppresses interferon-γ signaling in macrophages through a contact-dependent, gingipain-mediated mechanism

Shotaro Abe[1], Jun Ohshima[1,*], Masayoshi Morita[1], Nobutake Tanaka[1], Mikako Hayashi[1]

[1]Department of Restorative Dentistry and Endodontology, Graduate School of Dentistry, The University of Osaka, Suita, Osaka 565-0871, Japan

***Correspondence:**

Jun Ohshima DDS, PhD

Department of Restorative Dentistry and Endodontology

Graduate School of Dentistry, The University of Osaka,

1-8 Yamadaoka, Suita, Osaka 565-0871, Japan

Tel/Fax: +81 6 6879 2927

Email: ohshima.jun.dent@osaka-u.ac.jp

Supplementary Fig. 1

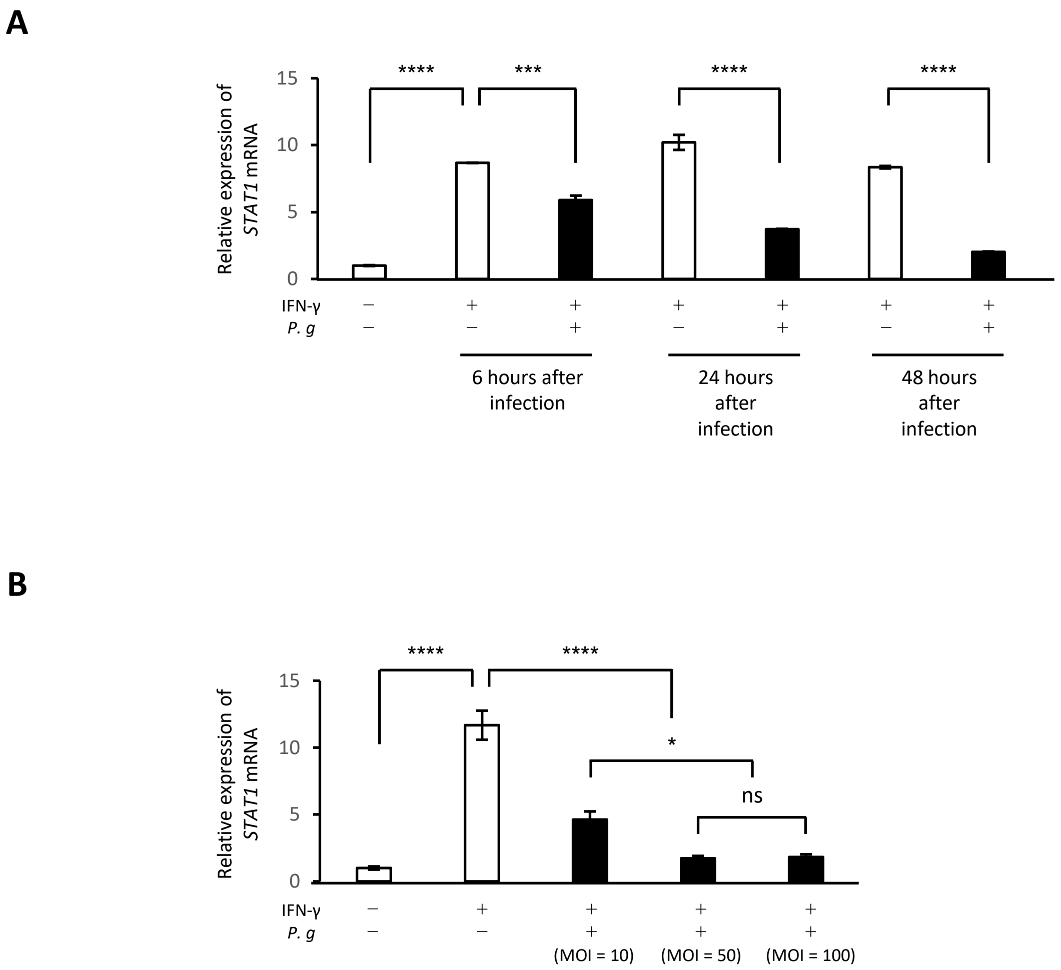

Supplementary Fig. 1

***P. gingivalis* infection reduces *STAT1* transcript abundance in a time- and dose-dependent manner.**

(A and B) THP-1 were infected with *P. gingivalis* at the times(A) and MOIs(B) indicated. *STAT1* mRNA levels were measured by qRT-PCR. Data were normalized to *GAPDH* mRNA and are expressed relative to non-infected controls. ns, not significant; * P < 0.05; **** P < 0.0001 (ANOVA with Tukey Test). All results are the mean ± SD and are representative of three independent experiments.

Supplementary Fig. 2

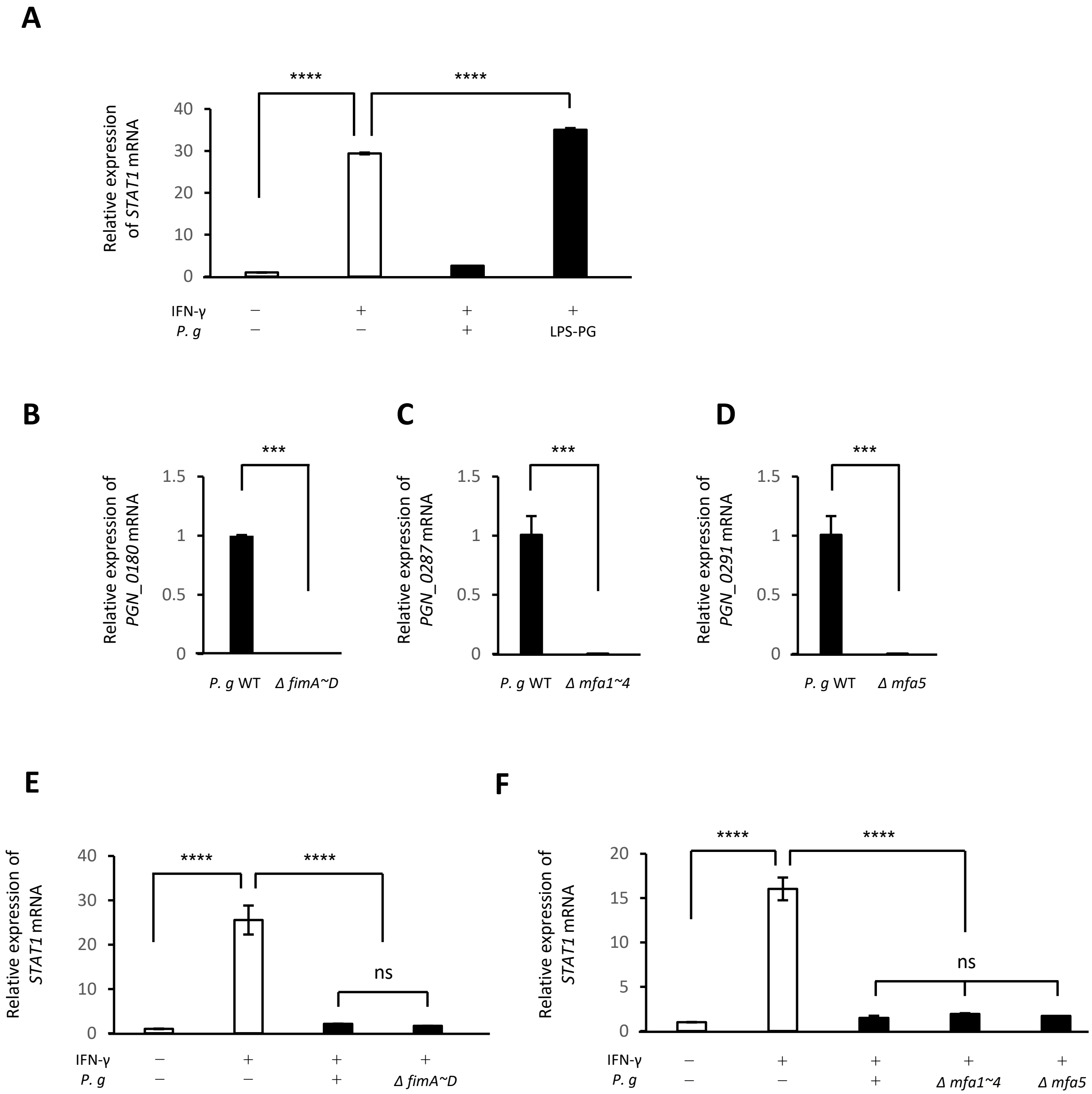

Supplementary Fig. 2

***P. gingivalis*-associated reduction in *STAT1* transcript levels is not mediated by LPS or fimbriae.**

(A) THP-1 were treated with PG-LPS (5 µg/mL). *STAT1* mRNA levels were measured by qRT-PCR. Data were normalized to *GAPDH* and are expressed relative to non-infected controls.

(B-D) Confirmation of deletion of *fimA* (B), *mfa1* (C), and *mfa5* (D) by qRT-PCR. Data were normalized to *P. gingivalis* 16S rRNA and are expressed relative to WT.

(E and F) THP-1 were infected with *P. gingivalis* Δ*fimA*(E), or Δ*mfa1-4* and Δ*mfa5*(F). *STAT1* mRNA levels were measured by qRT-PCR. Data were normalized to *GAPDH* and are expressed relative to non-infected controls.

All results are the mean ± SD and are representative of three independent experiments. ns, not significant; *** $P < 0.001$; **** $P < 0.0001$ (ANOVA with Tukey Test).

Supplementary Fig. 3

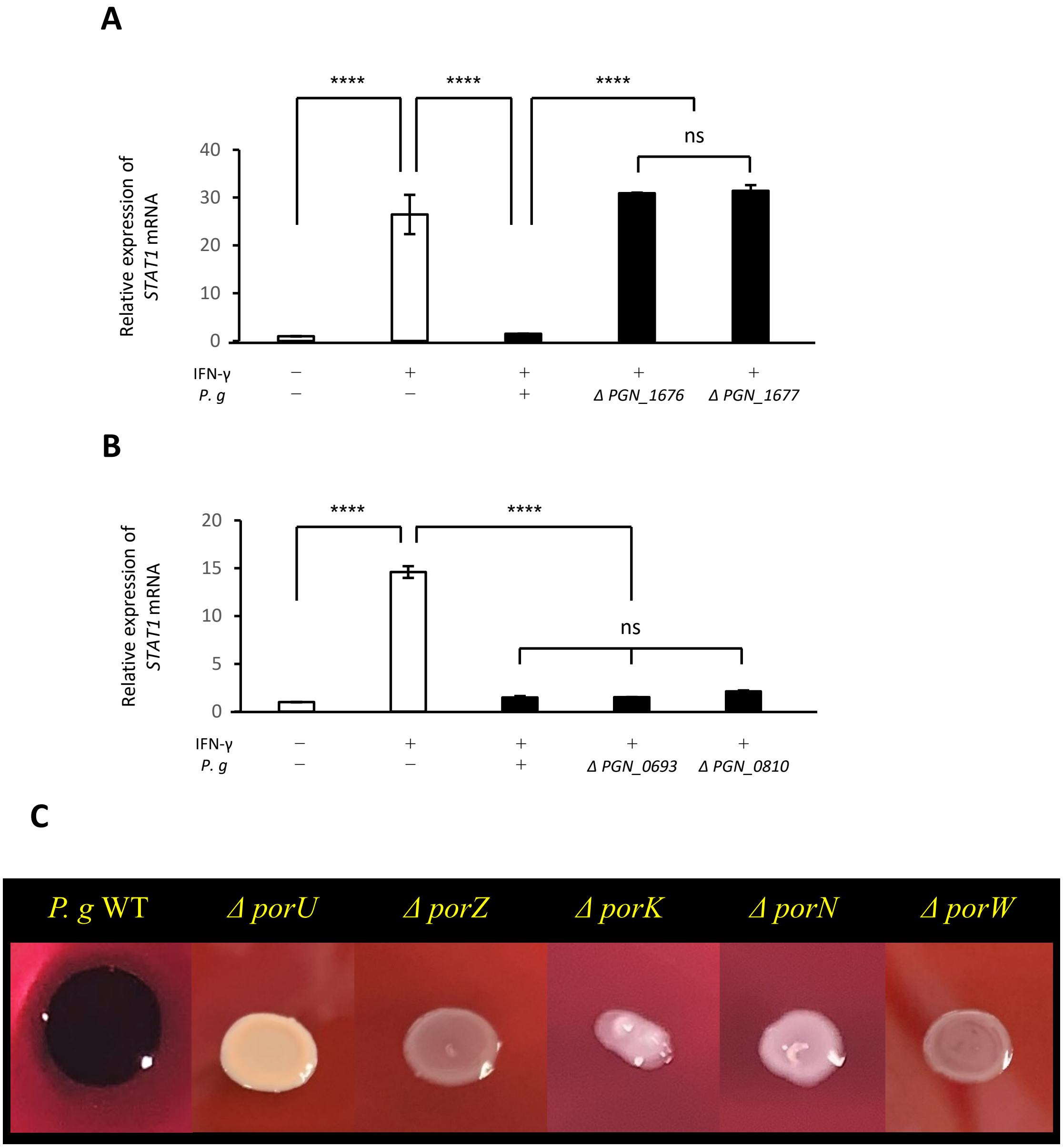

Supplementary Fig. 3

Role of T9SS-related proteins in *P*. *gingivalis*-mediated STAT1 suppression.
(A-B) THP-1 were infected with *P. gingivalis* Δ*polK,* Δ*porN* (A), Δ*PGN_0693* or Δ*PGN_0810* (B). *STAT1* mRNA levels were measured by qRT-PCR. Data were normalized to *GAPDH* mRNA and are expressed relative to non-infected controls. Results are the mean ± SD and are representative of three independent experiments. ns, not significant; **** $P < 0.0001$ (ANOVA with Tukey Test).
(C) Colony observation using blood agar medium of T9SS-related knockout strains.

Supplementary Fig. 4

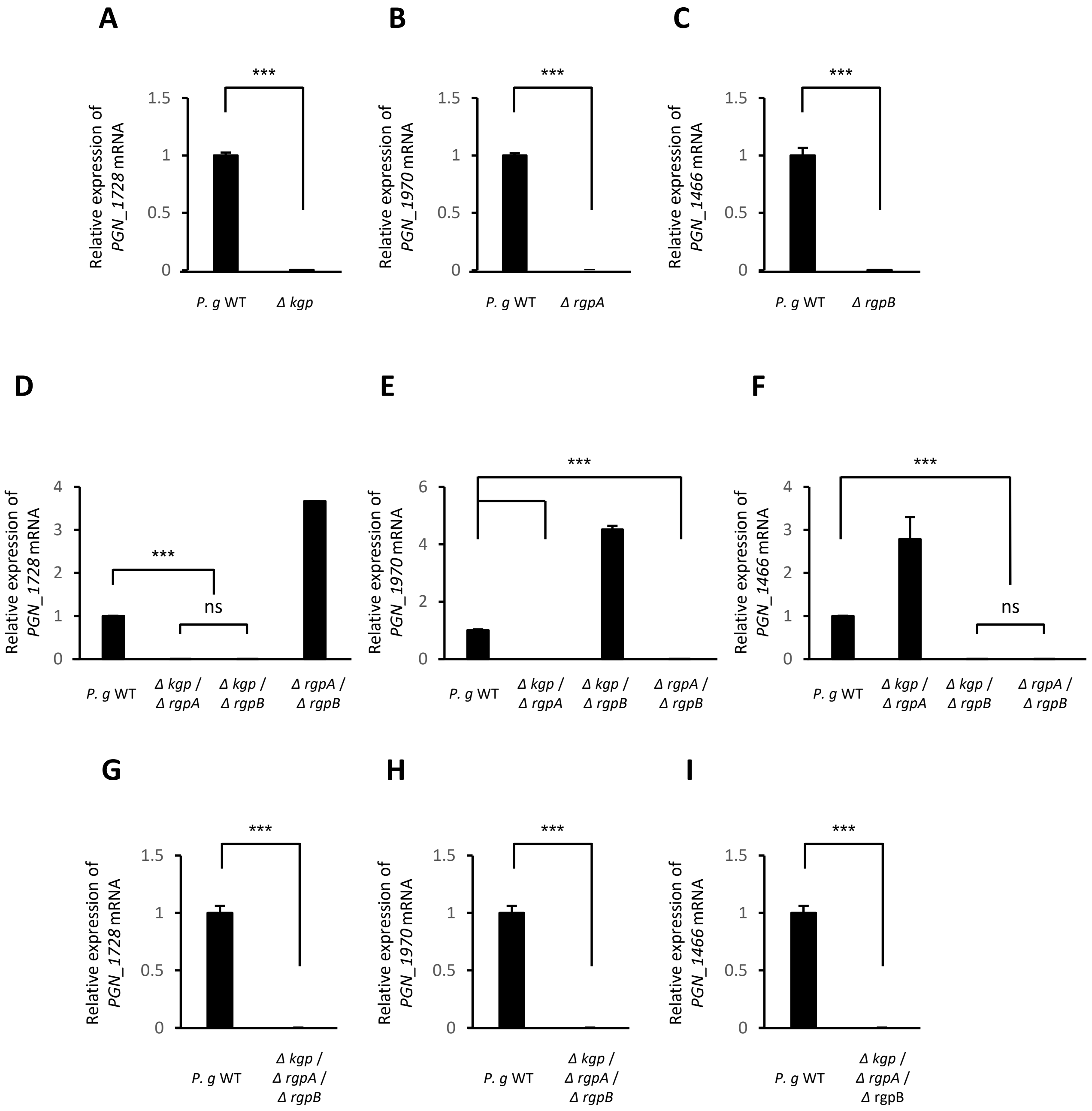

Supplementary Fig. 4

Role of gingipains in *P. gingivalis*-mediated suppression of *STAT1* expression.

(A-I) Confirmation of deletion of gingipain genes by RT-qPCR. Data were normalized to *P. gingivalis* 16S rRNA and are expressed relative to WT controls. All results are shown as mean ± SD and are representative of three independent experiments. ns, not significant; **** P < 0.0001 (ANOVA with Tukey Test).

Supplementary Fig. 5

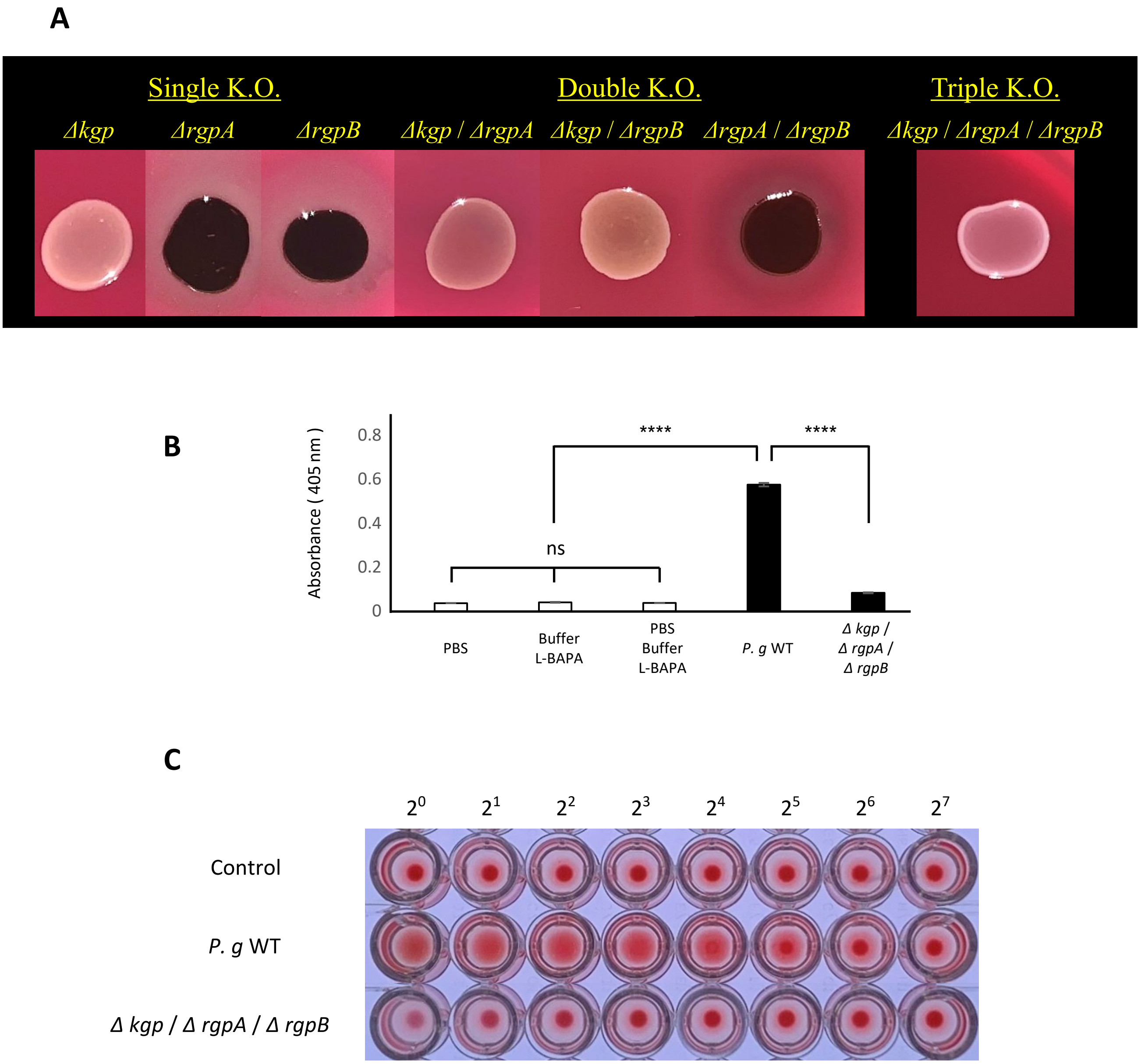

Supplementary Fig. 5

Functional validation of gingipain loss in the triple-deletion mutant.

(A) Colony growth of a gingipain knockout strain using blood agar medium.

(B) Measurement of gingipain activity in a triple-deletion mutant of gingipain genes using L-BAPA. Results are the mean ± SD and are representative of three independent experiments. ns, not significant; **** $P < 0.0001$ (ANOVA with Tukey Test).

(C) Hemagglutination reaction of gingipain triple-deficient mutants.

TABLES

Supplementary Table 1. List of bacterial strains

Strain	Characteristics	Locus tag
ATCC33277	Wild Type (*fimA* type I)	
	fimA-B-C-D::Em^r	PGN_0180-0184
	mfa1-2-3-4:: Em^r	PGN_0287-0290
	mfa5:: Em^r	PGN_0291
	PorU:: Em^r	PGN_0022
	PorZ:: Em^r	PGN_0509
	PorT:: Em^r	PGN_0778
	PorW:: Em^r	PGN_1877
	PGN_0693:: Em^r	PGN_0693
	PGN_0810:: Em^r	PGN_0810
	rgpA::Em^r	PGN_1970
	rgpB::Em^r	PGN_1466
	kgp::Em^r	PGN_1728
	rgpA::Em^r *rgpB*::Tc^r	PGN_1970, PGN_1466
	rgpA::Em^r *kgp*::Tc^r	PGN_1970, PGN_1728
	rgpB::Tc^r *kgp*::Em^r	PGN_1466, PGN_1728
	rgpA::Em^r *rgpB*::Tc^r *kgp*::Cm^r	PGN_1970, PGN_1466, PGN_1728
W50	Wild Type (*fimA* type IV)	
W83	Wild Type (*fimA* type IV)	

Supplementary Table 2. List of primers used for RT-qPCR

species	RT-qPCR Primer name	Sequence
Human	GAPDH_F	5’-GCTCAGACACCATGGGGAAG-‘3
	GAPDH_R	5’-GAACATGTAAACCATGTAGTTGAGG-‘3
	STAT1_F	5’-CCCAATCCAGATGTCTATGATC-‘3
	STAT1_R	5’-CTGAATATTCCCCGACTGAG-‘3
	GBP1_F	5’-CTATGAGGAACCGAGGAAGG-‘3
	GBP1_R	5’-CACGTTCCACTTCAATCTCC-‘3
	IL6_F	5’- AGACAGCCACTCACCTCTTCAG -‘3
	IL6_R	5’- TTCTGCCAGTGCCTCTTTGCTG -‘3
	OAS1_F	5’-AGGAAAGGTGCTTCCGAGGTAG-‘3
	OAS1_R	5’-GGACTGAGGAAGACAACCAGGT-‘3
P. gingivalis ATCC33277	*P. g* 16S_rRNA_F	5’-TGGGTTTAAAGGGTGCGTAG-‘3
	P. g 16S_rRNA_R	5’-CAATCGGAGTTCCTCGTGAT-‘3
	PGN_0180_F (FimA)	5’-AACAAAGACAACGAGGCAGAAC-‘3
	PGN_0180_R (FimA)	5’-CATCGCCAACTCCAAAAGCAC-‘3
	PGN_0183_F (FimC)	5’-ACTACTTCCACGCCTTCTC-‘3
	PGN_0183_R (FimC)	5’-TACTGTTGCCACTTCGTCC-‘3
	PGN_0184_F (FimD)	5’-TTGCCCTCCGATACTCTTCC-‘3
	PGN_0184_R (FimD)	5’-GCCTGCCATACCTTAGTTCC-‘3
	PGN_0185_F (FimE)	5’-GCAGAGAACAACCAAGTAACC-‘3
	PGN_0185_R (FimE)	5’-ATCCCTAATATCATCCCGCC-‘3
	PGN_0287_F (Mfa1)	5’-TCCTACCAGCAGCACTTTC-‘3
	PGN_0287_R (Mfa1)	5’-AGCATTTGCCAATTCGCC-‘3
	PGN_0291_F (Mfa5)	5’-GGTGCCTGCAAATAAGGGTA-‘3
	PGN_0291_R (Mfa5)	5’-GTCGACGGCAACGGGAACTA-‘3
	PGN_0022_F (PorU)	5’-CGGAGTCTCTGGTGGTTTTG-‘3
	PGN_0022_R (PorU)	5’-CTCCGGAGCAGAAGCCTGTG-‘3
	PGN_0509_F (PorZ)	5’-CCTCTTGCAGGCTCTTGTCG-‘3
	PGN_0509_R (PorZ)	5’-ACTCCGTTGGCTACGGCAAA-‘3
	PGN_0778_F (PorT)	5’-CCCCTACGCCGACTACAAAC-‘3
	PGN_0778_R (PorT)	5’-CACGCCAACGGAAAATCCGG-‘3

	PGN_1877_F (PorW)	5’-ACAGGCAGCAAGACAAGAG-‘3
	PGN_1877_R (PorW)	5’-AAAGCGAAAGGCGGAGAAG-‘3
	PGN_0693_F	5’-CCTCTGTCGTCTGTATTGTTCC-‘3
	PGN_0693_R	5’-TCGCATACCTCTGACAAGCC-‘3
	PGN_0810_F	5’-GGTTTATCCCAATCCGGTGAAG-‘3
	PGN_0810_R	5’-CCATAGAGAGCACCAAACGAC-‘3
	PGN_1970_F (RgpA)	5’-GCCGAGATTGTTCTTGAAGC-‘3
	PGN_1970_R (RgpA)	5’-AGGAGCAGCAATTGCAAAG-‘3
	PGN_1466_F (RgpB)	5’-CGCTGATGAAACGAACTTGA-‘3
	PGN_1466_R (RgpB)	5’-CTTCGAATACCATGCGGTT-‘3
	PGN_1728_F (Kgp)	5’-AGCTGACAAAGGTGGAGACCAAAGG-‘3
	PGN_1728_R (Kgp)	5’-TGTGGCATGAGTTTTTCGGAACCGT-‘3

Supplementary Table 3. List of primers used for mutant construction

Primer name	Enzyme	Sequence
PGN_0180-0184_UA_F (FimA-D)	*Mlu*I	5’-acgcgtACGAGATCATTGTACCCGAGGGAG-‘3
PGN_0180-0184_UA_R (FimA-D)	*Spe*I	5’-actagtCCATTTAGAGATTGTCTTGCATATAGCG-‘3
PGN_0180-0184_LA_F (FimA-D)	*Not*I	5’-gcggccgcTGTATGTGCTCATAGCATTCATGGCTG-‘3
PGN_0180-0184_LA_R (FimA-D)	*Xho*I	5’gtcgacGGTATGATCCACAGTCAGCATACCG-‘3
PGN_0287-0290_UA_F (Mfa1-4)	*Mlu*I	5’-acgcgtAAACGAGAAAATTTCGGCGCGT-‘3
PGN_0287-0290_UA_R (Mfa1-4)	*Spe*I	5’-actagtCCTCGTTATCACATATCCGAACCC-‘3
PGN_0287-0290_LA_F (Mfa1-4)	*Not*I	5’-gcggccgcGAAAATGGGAATTGATGGCATGCGC-‘3
PGN_0287-0290_LA_R (Mfa1-4)	*Xho*I	5’-ctcgagATCATTCGCATTTCCAGTTTCGCC-‘3
PGN_0291_UA_F (Mfa5)	*Mlu*I	5’-acgcgtATCAAGGAAGTGCTGCCGAAAGGCT-‘3
PGN_0291_UA_R (Mfa5)	*Spe*I	5’-actagtACAAATGGATCGAAACAACACGCGC-‘3
PGN_0291_LA_F (Mfa5)	*Not*I	5’-gcggccgcTTACCGATGGGGGTCTTTCGTTCGG-‘3
PGN_0291_LA_R (Mfa5)	*Xho*I	5’-ctcgagATTCGGAGGGATTCCCATAAGAGC-‘3
PGN_0022_UA_F (PorU)	*Mlu*I	5’-acgcgtGCAGCACCTCGAAGCCTATCTCGAA-‘3
PGN_0022_UA_R (PorU)	*Spe*I	5’-actagtCAGATCTCGTCCCGATTCTTTGGGC-‘3
PGN_0022_LA_F (PorU)	*Not*I	5’-gcggccgcACGGGTAGCTCTGAAGGGTAAGGTA-‘3
PGN_0022_LA_R (PorU)	*Xho*I	5’-ctcgagCTTCGGCTATTGTGCTACCACGATC-‘3

PGN_0509_UA_F (PorZ)	*Mlu*I	5’-acgcgtCATCGAAAGGCGGTTGTGTACGGAT-‘3
PGN_0509_UA_R (PorZ)	*Spe*I	5’-actagtCCGCCAAAGCACGATGACTGATTTG-‘3
PGN_0509_LA_F (PorZ)	*Not*I	5’-gcggccgcTTTGCTGCTGCTTCCGTAGCAGGAT-‘3
PGN_0509_LA_R (PorZ)	*Xho*I	5’-ctcgagCACGTATGTCCAGTTCTTGCCGTCG-‘3
PGN_0778_UA_F (PorT)	*Mlu*I	5’-acgcgtCACGATAGAGTCGTGCAAAGCATGC-‘3
PGN_0778_UA_R (PorT)	*Spe*I	5’-actagtGTAAGATCAACGCCTTATGCAGC-‘3
PGN_0778_LA_F (PorT)	*Not*I	5’-gcggccgcGGTCAGTGGCGCATATCCTTGCAAT-‘3
PGN_0778_LA_R (PorT)	*Xho*I	5’-ctcgagACCGGAATCTGACGCTCTATTCAGC-‘3
PGN_1877_UA_F (PorW)	*Mlu*I	5’-acgcgtACCGATATGACTTGCCGTCTCCTC-‘3
PGN_1877_UA_R (PorW)	*Spe*I	5’-actagtCATAGCGATCCAGTTCCTCATCGTC-‘3
PGN_1877_LA_F (PorW)	*Not*I	5’-gcggccgcGGGACGGGGAAATAAGAAAGAGAAGG-‘3
PGN_1877_LA_R (PorW)	*Xho*I	5’-ctcgagGTTCCAACATGGCACAGACAGC-‘3
PGN_0693_UA_F	*Mlu*I	5’-acgcgtGAACTCCTCTCCGGTCTTGATATTCG-‘3
PGN_0693_UA_R	*Spe*I	5’-actagtCTCCTCTCGATTCGTTTGTGGATGC-‘3
PGN_0693_LA_F	*Not*I	5’-gcggccgcCGAGAGGCACTTACATCGCAGAAA-‘3
PGN_0693_LA_R	*Xho*I	5’-ctcgagCTTTCGGCAATCTCCTGCATCACAG-‘3
PGN_0810_UA_F	*Mlu*I	5’-acgcgtCGGGTTTTCTCTGATCATAGGG-‘3
PGN_0810_UA_R	*Spe*I	5’-actagtTGCAGCCTGCAATTTGCCCCATAC-‘3
PGN_0810_LA_F	*Not*I	5’-gcggccgcCGAACGCTTCATCAAGCTCTGAT-‘3
PGN_0810_LA_R	*Xho*I	5’-ctcgagCATCGCAATCTCTCTTTCATCGCCTC-‘3
PGN_1970_UA_F (RgpA)	*Mlu*I	5’-acgcgtGCTCTTCCTTATTAGGAGGAATGGC-‘3
PGN_1970_UA_R	*Spe*I	5’-actagtCACATGAGAAACGACCGATGAAGAC-‘3

(RgpA)		
PGN_1970_LA_F (RgpA)	*Not*I	5’-gcggccgcGGCGTATCTCCGAAAGAGTGCGTAA-‘3
PGN_1970_LA_R (RgpA)	*Xho*I	5’-ctcgagCTACGTAAGACTTGCCGTCAACGAC-‘3
PGN_1466_UA_F (RgpB)	*Mlu*I	5’-acgcgtGGCAAATGCCCCAATATGGGAGAG-‘3
PGN_1466_UA_R (RgpB)	*Spe*I	5’-actagtAGAGCTGCTCCATTATGCTTTTCCC-‘3
PGN_1466_LA_F (RgpB)	*Not*I	5’-gcggccgcATAAGGGCTGTGCCGAGGAATCCAT-‘3
PGN_1466_LA_R (RgpB)	*Xho*I	5’-ctcgagCGATTCGTGCCTGCTATCGAAAGT-‘3
PGN_1728_UA_F (Kgp)	*Mlu*I	5’-acgcgtCTCAGCCGAGGAGCATACGGATATT-‘3
PGN_1728_UA_R (Kgp)	*Spe*I	5’-actagtATGGTCGTGATTCAGAGAACCACGG-‘3
PGN_1728_LA_F (Kgp)	*Not*I	5’-gcggccgcTGGACTCGGAGACTTTGTGCAGACA-‘3
PGN_1728_LA_R (Kgp)	*Xho*I	5’-ctcgagACGATTGAAGTCGCAGATGACAGGC-‘3
ErmF_F	*Spe*I	5’-actagtATGACAAAAAAGAAATTGCCCGTTCGT-‘3
ErmF_R	*Not*I	5’-gcggccgcTACGAAGGATGAAATTTTTCAGGGACAAC-‘3
TetQ_F	*Spe*I	5’-actagtATGAATATTATAAATTTAGGAATTCTTGCTCAC-‘3
TetQ_R	*Not*I	5’-gcggccgcTTATTTTGATGACATTGATTTTTGGAACATG-‘3
Cm_F	*Spe*I	5’-actagtATGGAGAAAAAAATCACTGGATATACCACCG-‘3
Cm_R	*Not*I	5’-gcggccgcTTACGCCCCGCCCTGCCACT-‘3

Supplementary Table 4. Top 30 differentially expressed genes in the IFN-γ group or IFN-γ + *P. gingivalis* group

**IFN-γ group**		**IFN-γ ＋ *P. gingivalis* group**	
Up Regulated Genes	Down Regulated Genes	Up Regulated Genes	Down Regulated Genes
CD74	HMGA2	CCL4	PSAT1
SERPING1	OLR1	CCL4L2	MX1
HLA-DRB1,HLA-DRB6	SERPINE2	CCL3L3	GBP5
MX1	LIF	CXCL8	DDIT4
GBP5	FN1	CCL3	PHGDH
HLA-DRA	MIR3917,STMN1	IL23A	ASNS
HLA-DRB5	GPNMB	CCL1	OAS1
C2,CFB,XXbac-BPG116M5.17	CCNB1	TNFAIP3	MX2
OAS1	DAPL1,OR7E28P,OR7E89P	CCL20	ISG15
OAS3	FBP1	POU2F2	OAS3
PARP14	CYP27A1	PIM2	IFITM3
STAT1	PTTG1	MARCKS	PLSCR1
CHI3L2	MLPH	BIRC3	OR56B1,TRIM22
GBP2,GBP7	COL22A1	TNF	IFIT3
IFITM3	NUSAP1	TRAF1	STAT1
IFIT3	CXCR4	IL1B	XAF1
PTK7	UBE2C	HCK	WARS
ISG15	SPP1	CD82	PARP9
OR56B1,TRIM22	LPL	AC021914.1,AMPD3	OAS2
MX2	LIPG	SOD2	PARP14
IFI6	NUF2	NFKBIA	GBP1
CTA-384D8.36	FMNL2	CD48	IFI35
XAF1	THBD	CD70	NXPH4
ODF3B,SCO2,TYMP	PCDH1	GM2A	NFIX
HLA-DPB1	EPB41L1	G0S2	SESN2

OAS2	AQP9	LINC00926	SLC7A5
S100A8	PCOLCE2	EBI3	ASS1
CASP1	SLA	CKB	CTA-384D8.36
GBP1	LPXN	IER3	IRF1
IL10RA	PLK1	GAL	PCK2

Supplementary Table 5. Genes encoding proteins secreted by the Type Ⅸ secretion system and anchored to the bacterial membrane

T9SS conserved C-terminal domain-containing proteins				Reference				
Strain	Locus tag	Symbol	Protein description	[1]	[2]	[3]	[4]	[5]
ATCC33277	PGN_0022	PorU	PorU; surface C-terminal sortase	○	○	○		○
	PGN_0123	PorA	Hypothetical protein		○	○	○	○
	PGN_0152	TapA	T9SS C-terminal target domain-containing protein; TapA, Immunoreactive 61 kDa antigen	○	○	○	○	○
	PGN_0291	Mfa5	Mfa5; VWA domain-containing protein [von Willebrand factor (vWF) type A domain]		○	○		○
	PGN_0295		C-terminal domain of Arg- and Lys-gingipain proteinase					○
	PGN_0335	CPG70	CPG70; zinc carboxypeptidase	○	○	○	○	○
	PGN_0352		conserved hypothetical protein					○
	PGN_0458		Uncharacterized protein	○				
	PGN_0509	PorZ	PorZ; surface B-propeller protein		○	○		○
	PGN_0561	PrtT	PrtT (Trypsin like proteinase PrtT); cystein protease (domain peptidase C10)	○	○			○
	PGN_0654		Hypothetical protein		○	○		○
	PGN_0657		Hypothetical protein		○	○		○
	PGN_0659	HBP35	HBP35 (hemin binding protein 35)		○	○	○	○
	PGN_0693		T9SS C-terminal target domain-containing protein	○	○	○	○	○

	PGN_ 0795		Fibronectin; hypothetical protein b		○	○		
	PGN_ 0810		T9SS C-terminal target domain-containing protein c	○	○	○		
	PGN_ 0852		T9SS C-terminal target domain-containing protein, leucine-rich repeats (x7), Immunoreactive 47 kDa antigen	○	○	○	○	○
	PGN_ 0898	PPAD	PPAD; peptidylarginine deiminase	○	○	○	○	○
	PGN_ 0900		Periodontain; peptidase C10; PrtT-related, Thiol protease	○	○	○		
	PGN_ 1115		Hemagglutinin		○	○		
	PGN_ 1317		Uncharacterized protein	○				○
	PGN_ 1321		T9SS C-terminal target domain-containing protein	○	○	○		○
	PGN_ 1416	PepK	PepK; lysine specific serine endopeptidase	○	○	○		○
	PGN_ 1466	RgpB	RgpB; arginine specific gingipain B, cysteine protease	○	○	○	○	○
	PGN_ 1476		T9SS C-terminal target domain-containing protein	○	○	○		○
	PGN_ 1556		T9SS C-terminal target domain-containing protein, leucine-rich repeats (x7), Putative hemagglutinin	○	○	○		○
	PGN_ 1611	SlrP	Internalin; hypothetical protein; leucine-rich repeats(x8)		○	○		
	PGN_ 1728	Kgp	Kgp; lysine specific gingipain, cysteine protease	○	○	○		○
	PGN_ 1733	HagA	HagA (hemagglutinin A, 8 HA domains)	○	○	○	○	○
	PGN_ 1767		T9SS C-terminal target domain-containing protein	○	○	○	○	○
	PGN_	P27	Hypothetical protein		○	○		○

	1770							
	PGN_ 1817		Uncharacterized protein	○				
	PGN_ 1970	RgpA	RgpA; arginine specific gingipain A; cysteine protease	○	○	○	○	
	PGN_ 2065		Hypothetical protein; peptidase, putative Lys- and Rgp- gingipain domain protein		○	○	○	○
	PGN_ 2080		Hypothetical protein		○	○	○	○

[1] Gabarrini, G., Grasso, S., van Winkelhoff, A. J., & van Dijl, J. M. (2020). Gingimaps: Protein Localization in the Oral Pathogen *Porphyromonas gingivalis*. *Microbiology and molecular biology reviews : MMBR*, *84*(1), e00032-19. https://doi.org/10.1128/MMBR.00032-19

[2] Lasica, A. M., Ksiazek, M., Madej, M., & Potempa, J. (2017). The Type IX Secretion System (T9SS): Highlights and Recent Insights into Its Structure and Function. *Frontiers in cellular and infection microbiology*, *7*, 215. https://doi.org/10.3389/fcimb.2017.00215

[3] Veith, P. D., Nor Muhammad, N. A., Dashper, S. G., Likić, V. A., Gorasia, D. G., Chen, D., Byrne, S. J., Catmull, D. V., & Reynolds, E. C. (2013). Protein substrates of a novel secretion system are numerous in the Bacteroidetes phylum and have in common a cleavable C-terminal secretion signal, extensive post-translational modification, and cell-surface attachment. Journal of proteome research, 12(10), 4449–4461. https://doi.org/10.1021/pr400487b

[4] Glew, M. D., Veith, P. D., Peng, B., Chen, Y. Y., Gorasia, D. G., Yang, Q., Slakeski, N., Chen, D., Moore, C., Crawford, S., & Reynolds, E. C. (2012). PG0026 is the C-terminal signal peptidase of a novel secretion system of Porphyromonas gingivalis. *The Journal of biological chemistry*, *287*(29), 24605–24617. https://doi.org/10.1074/jbc.M112.369223

[5] Gorasia, D. G., Veith, P. D., & Reynolds, E. C. (2020). The Type IX Secretion System: Advances in Structure, Function and Organisation. *Microorganisms*, *8*(8), 1173. https://doi.org/10.3390/microorganisms8081173
